# Availability and transparency of artificial intelligence models in radiology: a meta-research study

**DOI:** 10.1007/s00330-025-11492-6

**Published:** 2025-03-17

**Authors:** Taehee Lee, Jong Hyuk Lee, Soon Ho Yoon, Seong Ho Park, Hyungjin Kim

**Affiliations:** 1 Department of Radiology, Seoul National University Hospital, Jongno-gu, Korea; 2 Department of Radiology, Seoul National University College of Medicine, Jongno-gu, Korea; 3 Department of Radiology and Research Institute of Radiology, Asan Medical Center, University of Ulsan College of Medicine, Songpa-gu, Korea

**Keywords:** Artificial intelligence, Machine learning, Replicability, Reproducibility, Model availability

## Abstract

**Objectives:**

This meta-research study explored the availability of artificial intelligence (AI) models from development studies published in leading radiology journals in 2022, with availability defined as the transparent reporting of relevant technical details, such as model architecture and weights, necessary for independent replication.

**Materials and methods:**

A systematic search of Ovid Medline and Embase was conducted to identify AI model development studies published in five leading radiology journals in 2022. Data were extracted on study characteristics, model details, and code and model-sharing practices. The proportion of AI studies sharing their models was analyzed. Logistic regression analyses were employed to explore associations between study characteristics and model availability.

**Results:**

Of 268 studies reviewed, 39.9% (*n* = 107) made their models available. Deep learning (DL) models exhibited particularly low availability, with only 11.5% (*n* = 13) of the 113 studies being fully available. Training codes for DL models were provided in 22.1% (*n* = 25), suggesting limited ability to train DL models with one’s own data. Multivariable logistic regression analysis showed that the use of traditional regression-based models (odds ratio [OR], 17.11; 95% CI: 5.52, 53.05; *p* < 0.001) was associated with higher availability, while the radiomics package usage (OR, 0.27; 95% CI: 0.11, 0.65; *p* = 0.003) was associated with lower availability.

**Conclusion:**

The availability of AI models in radiology publications remains suboptimal, especially for DL models. Enforcing model-sharing policies, enhancing external validation platforms, addressing commercial restrictions, and providing demos for commercial models in open repositories are necessary to improve transparency and replicability in radiology AI research.

**Key Points:**

***Question*** *The study addresses the limited availability of AI models in radiology, especially DL models, which impacts external validation and clinical reliability*.

***Findings*** *Only 39.9% of radiology AI studies made their models available, with DL models showing particularly low availability at 11.5%*.

***Clinical relevance*** *Improving the availability of radiology AI models is essential for enabling external validation, ensuring reliable clinical application, and advancing patient care by fostering robust and transparent AI systems*.

**Graphical Abstract:**

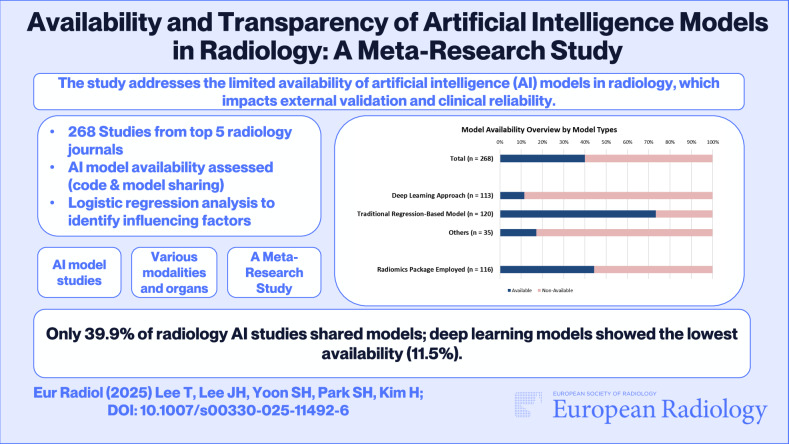

## Introduction

Recently, there has been a rapid increase in the publication of prediction models, including artificial intelligence (AI)-based ones, in radiology. To establish clinical utility, it is essential to assess these models for generalizability. This is typically achieved through external testing, which assesses whether models are prone to selection bias or overfitting. Selection bias occurs when the study population does not accurately represent the target population, limiting generalizability. Overfitting is another critical concern, where a model performs well on the training data but fails to generalize to unseen data due to overly complex patterns learned during training. One study found that nearly half of the deep learning (DL) models that underwent external testing showed at least a modest decrease in performance during external testing [1].

For effective external testing in the scientific community, models should be accessible to researchers. Guidelines such as the Checklist for Artificial Intelligence in Medical Imaging (CLAIM) and Must AI Criteria-10 advocate public code sharing to enhance transparency and reproducibility in AI research [2, 3]. Similarly, the CheckList for EvaluAtion of Radiomics Research for radiomics emphasizes the importance of disclosing both the code and the final model files [4]. Leading journals like *Radiology* [5], have mandated code sharing, and adherence to these practices is increasing [6].

Importantly, merely releasing the training code is insufficient for effective external testing by other researchers. The model architecture and weights must also be publicly available. Without original training data and hyperparameters, recreating the model with only the training code is impossible, and even with similar data, performance cannot be guaranteed. Moreover, there is a risk that the training code may not be fully disclosed. Therefore, reproducing results without access to the trained model is practically impossible. The lack of access to the model architecture and weights not only hinders replication and external testing but may also lead to redundant development of similar models, which does not foster progress in the scientific community [7]. The following example demonstrates successful model availability. Weise et al [8] published a DL model predicting 10-year cardiovascular risk from chest radiographs and facilitated replication by uploading sample images, preprocessing and inference code, and providing a download link for the model weights on their GitHub repository (https://github.com/circ-ml/CXR-CV-Risk). In contrast, some studies provide only partial resources, such as training code without inference code or model weights, which can limit replication.

In this study, we aimed to assess how frequently AI model development studies in radiology provided the technical and model details required for independent replication with a new dataset—what we defined as available models or model availability. Specifically, we investigated the proportion of studies published in leading radiology journals from January 1, 2022, to December 31, 2022, that shared models for potential replication.

## Materials and methods

This meta-research study was exempt from institutional review board approval and was registered prospectively on the International Prospective Register of Systematic Reviews (PROSPERO) network (registration number: CRD42024583225). The systematic review followed the Preferred Reporting Items for Systematic Reviews and Meta-Analyses (PRISMA) guidelines [9].

### Literature search and study selection

A systemic search of the Ovid Medline and Embase databases was conducted to identify articles on AI models published between January 1, 2022, and December 31, 2022, in *Radiology*, *Radiologia Medica*, *Investigative Radiology*, *European Radiology*, and *Diagnostic and Interventional Imaging*—the top five clinical radiology journals indexed in the Science Citation Index Expanded (SCIE) by the Journal Citation Reports impact factors in 2022 within the “Radiology, Nuclear Medicine, and Medical Imaging” category. In this review, AI models refer to a wide range of AI applications in radiology, encompassing both traditional statistical models and more recent DL approaches. The detailed search strategy is available in Supplementary Text 1 (online).

Studies were excluded if they (1) did not involve both the development and evaluation of AI models (e.g., studies that only tested a pre-existing AI algorithm); (2) solely conducted association analyses using regression statistics; (3) presented final models based on a single input variable; (4) were not original articles; and (5) were irrelevant to the research question. A full list of included articles is available in Supplementary Table 1 (online).

### Data extraction

The characteristics of studies, individuals, and models were systematically extracted: (a) study characteristics included journal of publication, country of the first author, image modality, and imaged body part; (b) individual characteristics included development set size (i.e., training, tuning, and internal test sets), whether external testing was conducted, and if so, size and source (categorized as different institution, open-access, or combined) of the external test set; (c) model characteristics included purpose of the model (i.e., diagnostic or prognostic), type of model (i.e., DL approaches, traditional regression-based models, or others), and use of radiomics packages (e.g., pyradiomics) for feature extraction.

Specifically, the development set size was categorized as: 1–99, 100–199, 200–499, 500–999, and 1000 or more individuals. A model was considered prognostic if its predictions involved a future event. A model was classified as using DL approaches if the final output was generated using methods such as convolutional neural networks, multilayer perceptrons, artificial neural networks, generative adversarial networks, or Transformers. A model was classified as a traditional regression-based model if the final output was generated through techniques such as logistic regression, Cox proportional hazards regression, linear regression, generalized linear models, or linear mixed-effects models. If multiple models were developed in a study but only one final model was presented, we focused on the final model.

### Primary analysis of model availability

In this meta-research study, a model was defined as being available if the study transparently reported relevant technical and model information for independent replication using a new dataset. For traditional regression-based models, availability was defined by sharing the model formula, nomogram, or selected predictors with their coefficients, odds ratios (OR), or hazard ratios [10]. DL models were considered available if both inference code and model with weights were accessible. If only model weights were accessible but the model used a standard DL architecture like U-Net, allowing inference without additional code, the model was still considered available. In cases where multiple models were developed for different purposes but only some were available, we documented this accordingly.

For DL studies, the availability of code for model training and inference was assessed, respectively. We verified code accessibility and documented the sharing platform (e.g., GitHub). We also recorded whether the shared code was for training, inference, or both. Any claims of code availability with broken links, empty repositories, or corrupted code were also documented.

The literature search, study selection, data extraction, and evaluation of model availability and code availability were performed by consensus between two board-certified radiologists (T.L. and H.K.) with expertise in AI model development and testing.

### Secondary analysis

Research artifact availability for replication requires both a model and consistent preprocessing. In the secondary analysis, a stricter definition of model availability was applied, considering a model available only if it met the primary analysis criteria and used replicable software during preprocessing. Studies were categorized based on the type of software used in preprocessing: (a) exclusive use of open-source software, (b) a combination of commercial and/or open-source software, or (c) in-house developed or unspecified software at any stage. However, software used exclusively during the training stage and not required for inference (e.g., solely needed for constructing ground truth) was excluded from the evaluation. We also considered radiomics packages used for feature extraction in this analysis, recognizing their role in research replication.

We evaluated software availability using three levels of definitions: (a) availability is limited to open-source software, (b) availability extends to both open-source and commercial software, and (c) all software, including in-house developed or unspecified software, are considered available.

Given that segmentation software tends to be more robust to the type of software used compared to other preprocessing tools, the secondary analysis was further divided into two parts:

1. General availability: this part considered the use of both segmentation and non-segmentation software during preprocessing, as well as radiomics packages for feature extraction.
2. Non-segmentation software and radiomics package only: assuming that segmentation software is inherently fully replicable due to its robustness, this part focused on the availability of non-segmentation software and radiomics packages.

We first conducted the analysis on all AI models, followed by a subgroup analysis of DL studies to evaluate the impact of preprocessing software and radiomics packages on research replication.

### Statistical analysis

Descriptive statistics were used to summarize the characteristics of the articles, the availability of models across all studies, and specifically for DL models. Univariable and multivariable logistic regression analyses were performed to evaluate the associations of model availability with various factors, including the journal of publication; image modality; imaged body part; development set size; external testing status; model purpose (diagnostic vs. prognostic); model type (DL approaches, traditional regression-based models, or others) and radiomics packages usage.

Statistical analyses were conducted using the R software version 3.5.1 (R Foundation for Statistical Computing, Vienna, Austria) with *p* < 0.05 showing statistical significance.

## Results

### Literature search

**Fig. 1:**
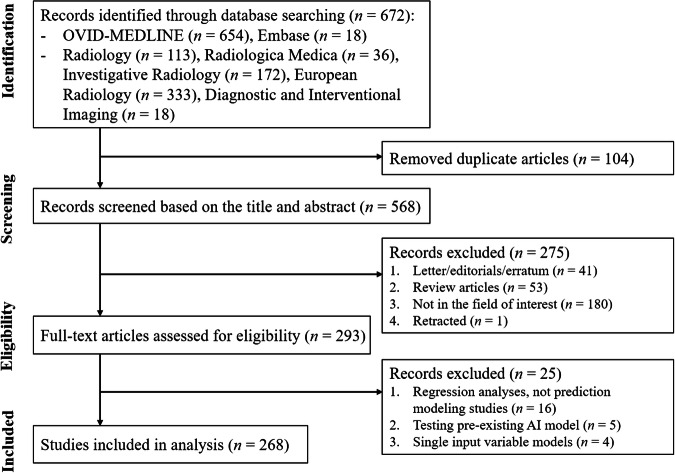
Flowchart of the study selection process

The study selection process is described in Fig. 1. We identified 672 articles from the initial systematic literature. After removing 104 duplicates, screening of the 568 titles and abstracts yielded 293 potentially eligible studies. After full-text reviews, 25 studies were excluded for the following reasons: (a) regression analyses, not prediction modeling studies (*n* = 16); (b) testing of pre-existing AI models (*n* = 5); and (c) single-input variable models (*n* = 4). Consequently, a total of 268 articles were included in the current meta-research.

### Study characteristics

Table 1 summarizes the study characteristics. Most studies were published in *European Radiology* (73.9%, 198/268) and *Radiology* (12.3%, 33/268). The majority originated from Asia (61.9%, 166/268) and Europe (26.5%, 71/268). Cross-sectional imaging modalities (CT, MRI, and PET/PET-CT) were predominant (82.5%, 221/268). The studies focused on thoracic, cardiovascular, and breast imaging (34.7%, 93/268) and abdominal imaging (28.0%, 75/268).

**Table 1:** Characteristics and model availability of the included studies

Characteristics	Total number of studies (*n* = 268)	Number of studies with available models (*n* = 107)	Percentage of studies with available models
Journal			
* Radiology*	33 (12.3)	11 (10.3)	33.3%
*Radiologia Medica*	20 (7.5)	13 (12.1)	65.0%
*Investigative Radiology*	12 (4.5)	3 (2.8)	25.0%
*European Radiology*	198 (73.9)	79 (73.8)	39.9%
*Diagnostic and Interventional Imaging*	5 (1.9)	1 (0.9)	20.0%
Country of the first author			
Asian Countries	166 (61.9)	71 (66.4)	42.8%
European Countries	71 (26.5)	26 (24.3)	33.6%
North American Countries	24 (9.0)	6 (5.6)	25.0%
Australia	1 (0.4)	1 (0.9)	100.0%
Multi-country entries	6 (2.2)	3 (2.8)	50.0%
Image modality			
Cross-sectional imaging (CT, MR, PET/PET-CT)	221 (82.5)	92 (86.0)	41.7%
Projection-based imaging (X-ray/MG/DBT)	21 (7.8)	3 (2.8)	14.3%
Ultrasound (US)	15 (5.6)	6 (5.6)	40.0%
Multimodality	11 (4.1)	6 (5.6)	54.5%
Imaged body part			
Thorax, cardiovascular, and breast	93 (34.7)	31 (29.0)	33.3%
Abdomen	75 (28.0)	38 (35.5)	50.7%
Neuro, head, and neck	68 (25.4)	28 (26.2)	41.2%
Musculoskeletal	24 (9.0)	5 (4.7)	20.8%
Multiple regions	8 (3.0)	5 (4.7)	62.5%
Size of development set			
1–99	47 (17.5)	19 (17.8)	40.4%
100–199	71 (26.5)	35 (32.7)	49.3%
200–499	80 (29.9)	36 (33.6)	45.0%
500–999	34 (12.7)	11 (10.3)	32.4%
1000~	36 (13.4)	6 (5.6)	16.7%
External testing conducted	79 (29.5)	32 (29.9)	40.5%
Different institution	60 (75.9)	27 (84.4)	45.0%
Open-access resource	10 (12.7)	4 (12.5)	40.0%
Combined sources or not specified	9 (11.4)	1 (3.1)	11.1%
Model purpose			
Diagnostic	166 (61.9)	48 (44.9)	28.9%
Prognostic	102 (38.1)	59 (55.1)	57.8%
Model type			
DL approach	113 (42.2)	13 (12.1)	11.5%
Traditional regression-based model	120 (44.8)	88 (82.2)	73.3%
Others	35 (13.1)	6 (5.6)	17.1%
Radiomics package employed	106 (39.6)	47 (43.9)	44.3%

Data in parentheses are percentages. Availability is defined by whether the study transparently reported relevant technical and model information for independent replication using a new dataset. For DL models, both inference code and model with weights must be accessible. For traditional regression-based models, availability was determined by sharing the model formula, predictors, and their coefficients or odds/hazard ratios

Regarding development set size, 56.3% (151/268) of studies used medium-sized development sets (100–499 individuals). External testing was conducted in 29.5% (79/268) of studies, with most datasets sourced from different institutions (75.9%, 60/79), followed by open-access resources (12.7%, 10/79). Diagnostic models accounted for 61.9% (166/268), while prognostic models made up 38.1% (102/268). DL approaches were employed in 42.2% (113/268), radiomics packages in 39.6% (106/268), and traditional regression-based models in 44.8% (120/268).

**Fig. 2:**
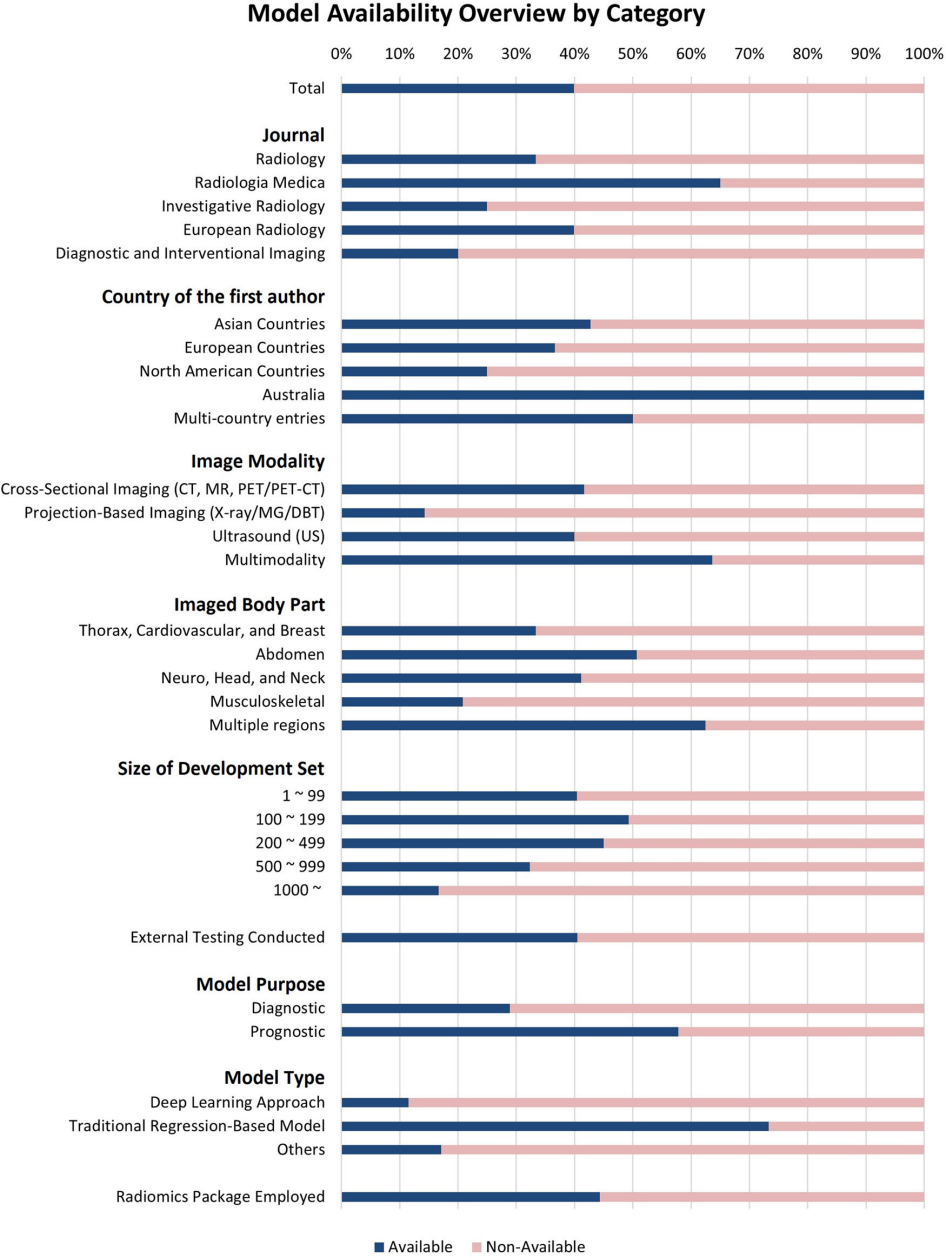
Model availability overview by category

Figure 2 illustrates the distribution of studies with and without available models across categories.

### Primary analysis of model availability

**Fig. 3:**
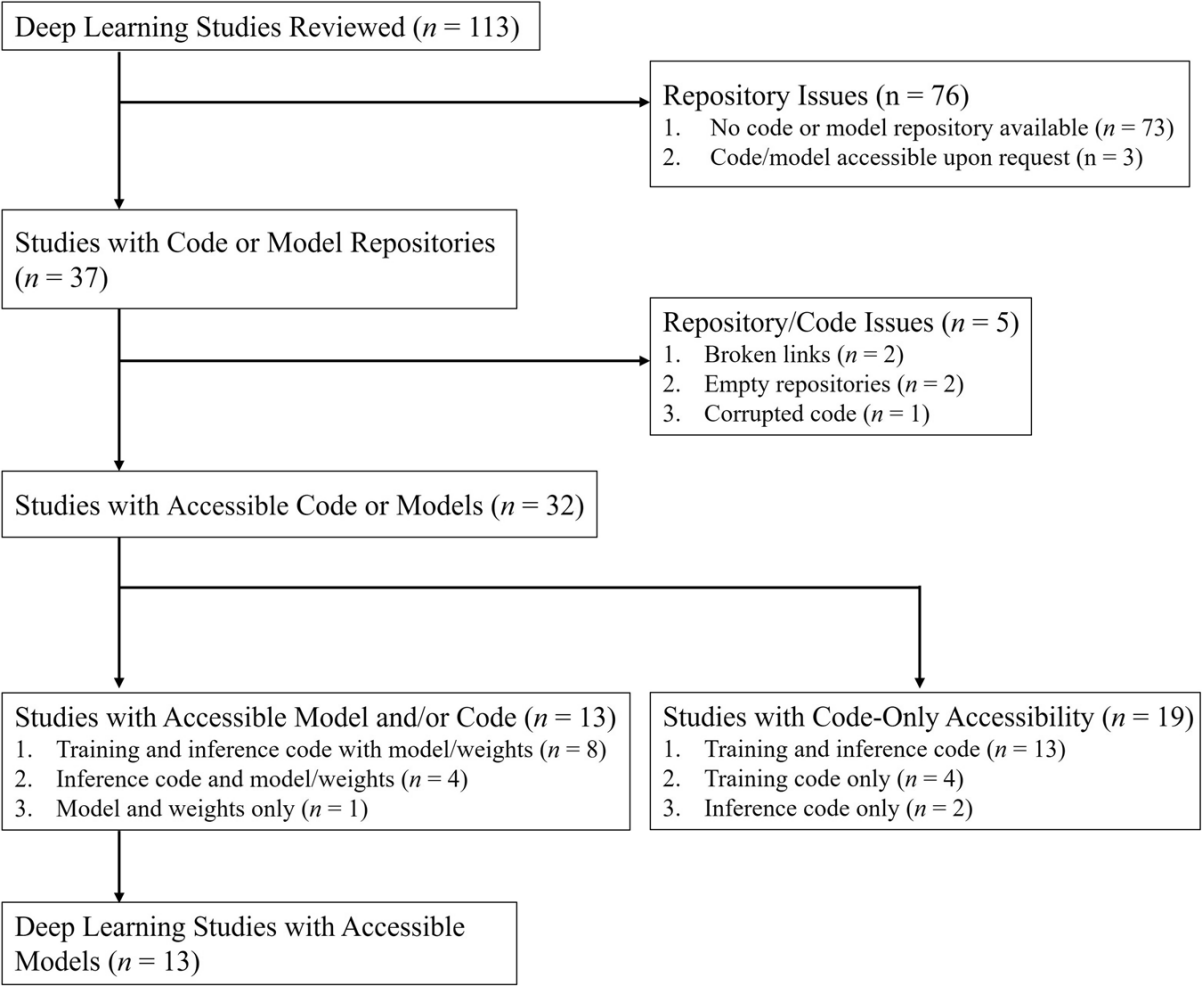
Flowchart of code and model accessibility in DL studies

Of the 268 studies, 107 (39.9%) made their models available (Table 1). Traditional regression-based models had the highest availability rate (73.3%, 88/120), followed by radiomics models (44.3%, 47/106). DL models had the lowest availability rate at 11.5% (13/113) (Table 2). Of the 13 available DL models, 12 provided inference code and models with weights, while one shared only the model without code (Fig. 3). Nevertheless, this study was still considered to have made the model available, as it used standard architectures, enabling straightforward inference without specific guides or codes. One of the DL studies claimed that their trained networks were available upon request but did not upload their models to a repository [11]. While the authors actively responded to our request and clarified that access to the trained networks requires a research model sharing agreement, this agreement imposes additional restrictions, including usage limitations and compliance with EU regulations. These requirements introduce some uncertainty regarding the model availability. Therefore, the model in this study was not classified as available in our analysis. If this study is considered to have provided an available model, then 12.4% (14/113) of the DL studies could be deemed available. Besides model availability, training codes for DL models were provided in 22% (25/113) of the studies, suggesting the limited ability to train DL models with one’s own data.

**Table 2:** Characteristics and model availability of DL studies

Characteristics	Total no. of DlL studies (*n* = 113)	Number of DL studies with available models (*n* = 13)	Percentage of studies with available models
Journal			
*Radiology*	21 (18.6)	2 (15.4)	9.5%
*Radiologia Medica*	3 (2.7)	0 (0.0)	0.0%
*Investigative Radiology*	11 (9.7)	3 (23.1)	27.3%
*European Radiology*	75 (66.4)	8 (61.5)	10.7%
*Diagnostic and Interventional Imaging*	3 (2.7)	0 (0.0)	0.0%
Image modality			
Cross-sectional imaging (CT, MR, PET/PET-CT)	87 (77.0)	12 (92.3)	13.8%
Projection-based imaging (X-ray/MG/DBT)	17 (15.0)	0 (0.0)	0.0%
Ultrasound (US)	8 (7.1)	0 (0.0)	0.0%
Multimodality	1 (0.9)	1 (7.7)	100%
Imaged body part			
Thorax, cardiovascular, and breast	45 (39.8)	5 (38.5)	11.1%
Abdomen	20 (17.7)	1 (7.7)	5.0%
Neuro, head, and neck	30 (26.5)	5 (38.5)	16.7%
Musculoskeletal	14 (13.4)	0 (0.0)	0.0%
Multiple regions	4 (3.5)	2 (15.4)	50.0%
Size of development set			
1–99	14 (12.4)	1 (7.7)	7.1%
100–199	18 (15.9)	1 (7.7)	5.6%
200–499	36 (31.9)	5 (38.5)	13.9%
500–999	15 (13.3)	2 (15.4)	13.3%
1000~	30 (26.5)	4 (30.8)	13.3%
External testing conducted	46 (40.7)	8 (61.5)	17.4%
Model purpose			
Diagnostic	88 (77.9)	9 (69.2)	10.2%
Prognostic	25 (22.1)	4 (30.8)	16.0%
Radiomics package employed	18 (15.9)	1 (7.7)	5.6%

Data in parentheses are percentages

*DL* deep learning

The model availability varied by journals. *Radiologia Medica* had the highest rate (65.0%, 13/20) (Table 1), while *European Radiology* demonstrated 39.9% (79/198) and *Radiology* had a lower rate of 33.3% (11/33). Cross-sectional imaging studies had an availability rate of 41.7% (92/221), whereas projection-based imaging had the lowest at 14.3% (3/21). Abdominal imaging studies were more available (50.7%, 38/75) compared to musculoskeletal imaging studies (20.8%, 5/24). Prognostic models were more available (57.8%, 59/102), compared to diagnostic models (28.9%, 48/166). Of the 107 studies with available models, three developed multiple models, but only one model in each was shared.

### Secondary analysis

When the analysis was limited to studies using open-source or commercial software for preprocessing, the proportion of available models slightly decreased to 37.3% (100/268) (Table 3). When further restricted to studies using only open-source software, this number dropped to 23.5% (63/268). In a separate analysis focused on non-segmentation software and radiomics packages—where segmentation software was considered replicable—the proportion of available models was 39.6% (106/268) with open-source or commercial software. This further declined to 31.7% (85/268) with only open-source software.

**Table 3:** Secondary analysis of model availability based on software used in preprocessing and radiomics package

Software availability criteria	Number of available models (all models) (*n* = 268)	Number of available models (DL models) (*n* = 113)
General availability (all software)		
Only open-source software is considered available	63 (23.5)	11 (9.7)
Both open-source and commercial software are considered available	100 (37.3)	13 (11.5)
All software, including in-house developed or unspecified software, is considered available	107 (39.9)	13 (11.5)
Non-segmentation Software and Radiomics Package Only (with segmentation considered replicable)		
Only open-source software is considered available	85 (31.7)	11 (9.7)
Both open-source and commercial software are considered available	106 (39.6)	13 (11.5)
All software, including in-house developed or unspecified software, is considered available	107 (39.9)	13 (11.5)

Data in parentheses are percentages. In this secondary analysis, a model was considered available if the study transparently reported relevant technical and model information, along with the requirement of using available software during preprocessing

The proportion of available models in the DL studies remained at 11.5% (13/113) for those using open-source or commercial software but dropped slightly to 9.7% (11/113) when restricted to studies using only open-source software. This trend was consistently observed in studies involving non-segmentation software and radiomics packages.

### Factors associated with model availability

Univariable logistic regression showed that studies in *Radiologia Medica* (OR, 3.71; 95% confidence interval (CI): 1.15, 11.96; *p* = 0.028), abdominal imaging (OR, 2.05; 95% CI: 1.10, 3.84; *p* = 0.024), prognostic models (OR, 3.28; 95% CI: 1.96, 5.49; *p* < 0.001), and traditional regression-based models (OR, 13.29; 95% CI: 5.05, 34.98; *p* < 0.001) had higher availability (Table 4). In contrast, projection-based imaging (OR, 0.23; 95% CI: 0.07, 0.82; *p* = 0.023) and larger sets (≥ 1000 individuals, OR, 0.30; 95% CI: 0.10, 0.84; *p* = 0.023) were associated with lower availability. In multivariable analysis, traditional regression-based models remained associated with higher availability (OR, 17.11; 95% CI: 5.52, 53.05; *p* < 0.001), while no factors for lower availability remained significant. Additionally, external testing (OR, 2.51; 95% CI: 1.09, 5.76; *p* = 0.029) was associated with higher availability, while the use of radiomics packages (OR, 0.27; 95% CI: 0.11, 0.65; *p* = 0.003) was associated with lower availability. However, these associations were significant only in the multivariable analysis.

**Table 4:** Logistic regression of model availability

		Univariable analysis		Multivariable analysis	
Characteristics	No. of studies included	OR	*p* value	OR	*p* value
Journal (reference: *Radiology*)					
*Radiologia Medica*	20	3.71 [1.15–11.96]	0.028^*^	4.36 [0.76–25.08]	0.10
*Investigative Radiology*	12	0.67 [0.15–2.97]	0.59	3.13 [0.42–23.46]	0.27
*European Radiology*	198	1.33 [0.61–2.89]	0.47	1.03 [0.32–3.26]	0.96
*Diagnostic and Interventional Imaging*	5	0.50 [0.05–5.03]	0.56	0.87 [0.04–18.79]	0.93
Modality (reference: cross-sectional imaging)					
Projection-based imaging	21	0.23 [0.07–0.82]	0.023^*^	0.73 [0.15–3.54]	0.69
Ultrasound	15	0.94 [0.32–2.72]	0.90	0.73 [0.14–3.82]	0.71
Multimodality	11	1.68 [0.50–5.68]	0.40	0.50 [0.10–2.47]	0.40
Imaged body part (reference: thorax, cardiovascular, and breast)					
Abdomen	75	2.05 [1.10–3.84]	0.024^*^	1.01 [0.40–2.55]	0.99
Neuro, head, and neck	68	1.40 [0.73–2.68]	0.31	1.49 [0.58–3.84]	0.41
Musculoskeletal	24	0.53 [0.18–1.54]	0.24	0.63 [0.15–2.59]	0.52
Multiple regions	8	3.33 [0.75–14.86]	0.11	8.60 [1.08–68.35]	0.042^*^
Size of development set (reference: 1–99)					
100–199	71	1.43 [0.68–3.02]	0.34	2.66 [0.92–7.69]	0.070
200–499	80	1.21 [0.58–2.50]	0.62	4.27 [1.44–12.62]	0.009^*^
500–999	34	0.71 [0.28–1.78]	0.46	1.94 [0.48–7.85]	0.35
1000~	36	0.30 [0.10–0.84]	0.023^*^	1.81 [0.39–8.52]	0.45
External testing conducted	79	1.04 [0.61–1.77]	0.90	2.51 [1.09–5.76]	0.029^*^
Model purpose (reference: diagnostic)					
Prognostic	102	3.28 [1.96–5.49]	< 0.001^*^	1.81 [1.02–3.94]	0.10
Model type (reference: others)					
DL approach	113	0.63 [0.22–1.80]	0.39	0.29 [0.08–1.08]	0.066
Traditional regression-based model	120	13.29 [5.05–34.98]	< 0.001^*^	17.11 [5.52–53.05]	< 0.001^*^
Radiomics package employed	106	1.32 [0.80–2.17]	0.28	0.27 [0.11–0.65]	0.003^*^

Data in parentheses are 95% CIs. Availability is defined by whether the study transparently reported relevant technical and model information for independent replication using a new dataset. For DL models, both inference code and model with weights must be accessible. For traditional regression-based models, availability was determined by sharing the model formula, predictors, and their coefficients or odds/hazard ratios

*OR* odds ratio

^*^ Statistically significant difference

## Discussion

Public sharing of AI models is essential for reproducible research and external testing, allowing researchers to verify results and assess model generalizability across diverse settings. This openness fosters scientific integrity, encourages collaboration, and enhances the development of robust AI systems. Our study aimed to evaluate the availability of models in AI studies published in leading radiology journals. Our findings showed that 39.9% (107/268) of studies shared their models. Limiting availability to studies using open-source software for preprocessing and radiomics packages, this figure dropped to 23.5% (63/268). For DL models, availability was even lower at 11.5% (13/113), and further restricting the analysis to models using only open-source software for preprocessing reduced this proportion to 9.7% (11/113).

Traditional regression-based models showed notably higher availability (73.3%, 88/120) than DL models (11.5%, 13/113). This is likely due to the ease of presenting models through coefficients, ORs, hazard ratios, regression formulas, and nomograms.

Among the journals examined, none required the disclosure or sharing of AI models. However, *Radiology* was the only one mandating code disclosure [12], which led to 66.7% (14/21) of DL studies sharing their code. Despite this, without a requirement to disclose models with weights, the model availability in *Radiology* was only 9.5% (2/21). While stronger model-sharing policies, particularly for DL studies, could enhance availability, such policies alone may not be sufficient to increase the number of available models. Stricter requirements might lead some developers to favor preprint platforms over peer-reviewed journals to avoid sharing models, especially with commercial interests.

Additionally, model-sharing practices are often influenced by institutional and regulatory constraints, such as data protection requirements, usage restrictions, and distribution controls (e.g., MDR/FDA regulations). These factors are particularly pertinent for trained models, which may incorporate sensitive data or present privacy risks, such as the theoretical potential for adversarial attacks enabling reidentification. Furthermore, commercial interests may deter full disclosure of models and weights due to concerns over intellectual property protection.

Given these challenges, alternative options, such as providing a user-accessible demo or web-based interfaces, could serve as practical solutions to balance transparency with these constraints [13, 14]. For instance, Bercean et al reported on a commercial AI system for COVID-19 volumetric quantification and included a demo webpage (https://rayscape.ai/lung-ct) in their publication [15]. In cases where sharing models or code is not feasible, requiring authors to explicitly state the reasons for non-disclosure could improve transparency and promote sharing. To further address these issues, Table 5 summarizes the essential reporting items for AI research artifacts availability, with a focus on data, code, and model sharing. This table also aligns these key aspects with the CLAIM 2024 checklist, providing a structured framework to improve artifact availability in AI research and ensure transparency.

**Table 5:** Essential reporting items for AI research artifacts availability

Category	Essential item	Description	Related CLAIM items (CLAIM 2024 version)
Data	Data availability	Indicate if training and testing data are publicly available, specify access methods (e.g., open-access links), and include any restrictions on usage.	Item 7 (data sources)
	Preprocessing details	Specify the software (with versions), step-by-step description of preprocessing steps, and all option and configuration settings used (e.g., normalization parameters and augmentation types).	Item 9 (data preprocessing)
Model	Code sharing (DL)	Indicate if training and inference code are shared, with links if available.	Item 22 (detailed description of the model), 43 (statement about the availability of software, trained model, and/or data*)*
	Model weights availability (DL)	Specify if trained model weights are publicly accessible.	Item 43
	Model formula and parameters (regression)	Share model formula, selected predictors with coefficients, ORs, HRs, and visual tools like nomograms.	N/A (CLAIM 2024 does not explicitly address regression models)
	Alternative options for non-disclosure	If code or model is not shared, specify alternative options (e.g., demo interfaces) or provide reasons for non-disclosure (e.g., ethical, regulatory, or commercial considerations).	Item 40 (study limitations), 43

Recent studies have highlighted challenges in achieving generalizability and reproducibility of AI models in radiology. A comprehensive review [16] examining AI studies in radiology published between 2015 and 2019 found that external testing was conducted in 32% (169/535), with a 6% average performance decrease. Another systemic review [17] of AI models for head CT scans revealed that only 8% (7/83) provided open-source code, hindering replication. A third systematic review [18], exploring DL studies for medical imaging published in the *Journal of Digital Imaging*, found that 5% (4/80) shared code, 44% (35/80) released their datasets or used open-access data, and 6% (5/80) performed external testing. A fourth systemic review [19] of machine learning algorithms for medical imaging, excluding traditional statistical methods, revealed that raw data was accessible in 18% (34/194). About one-tenth of the studies made their pre-modeling (13%, 25/194), modeling (14%, 28/294), or post-modeling files (8%, 15/194) accessible. Additionally, a meta-research study [6] on AI studies published in the Radiological Society of North America (RSNA) journals suite from 2017 to 2021 showed that 34% (73/218) shared code, but only 11% (24/218) shared replicable code.

In addition to the existing body of evidence, the merit of our study lies in its comprehensive investigation of AI model replicability across a broad spectrum of approaches. This includes not only DL but also models such as logistic regression, support vector machines, and tree-based models. Our study covered leading clinical radiology journals without restricting the scope, providing a holistic understanding of model availability. Specifically focusing on DL studies, this is the first research to emphasize model availability from the perspectives of external testing and replication, assessing whether both the model architecture and weights are being shared. Additionally, our study examined the preprocessing and feature extraction steps and conducted secondary analyses using multiple stricter definitions of model availability.

Our study had limitations. First, the research focused on articles published in 2022, which may not reflect recent trends. Second, the study focused on five leading clinical radiology journals, limiting generalizability to other types of journals, such as *Medical Image Analysis* or *IEEE Transactions on Medical Imaging* (excluded due to their engineering orientation) and *Radiology: Artificial Intelligence* (excluded as it was not indexed in the SCIE). Follow-up studies should cover these journals and updated policies. Third, we did not evaluate whether the environment required to run the code was sufficiently described, whether the code functioned without errors, or whether the results matched those reported.

In conclusion, the availability of AI models in radiology remains limited, especially for DL models. To improve transparency and availability in radiology AI research, it is essential to enforce stricter model-sharing policies, enhance external validation platforms, address commercial restrictions, and provide demos for commercial models in open repositories.

## Supplementary information

Electronic Supplementary Material

## Abbreviations

AI: Artificial intelligence
CI: Confidence interval
DL: Deep learning
OR: Odds ratio
