# Supplementary material for: Availability and transparency of artificial intelligence models in radiology: a meta-research study
Source: Eur Radiol. 2025 Mar 17;35(9):5287–98. doi: 10.1007/s00330-025-11492-6 (PMC12350510; doi:10.1007/s00330-025-11492-6)
Supplement: Supplementary file 1 — Electronic Supplementary Material [file 330_2025_11492_MOESM1_ESM.pdf]

Electronic Supplementary Material

Supplementary Text 1: LITERATURE DATABASE SEARCH

A systematic search was conducted using the OVID-MEDLINE and Embase databases. The search included terms related to artificial intelligence (AI), including *AI*, *deep learning*, *machine learning*, *artificial intelligence*, *neural net**, and *model*. The search was further restricted to studies published in the following radiology journals: *Radiology, Radiologia Medica, Investigative Radiology, European Radiology, and Diagnostic and Interventional Imaging*. The final search strategy combined these two categories, and the publication year was limited to 2022.

The OVID-MEDLINE search strategy included:

- Keywords related to artificial intelligence: "AI," "deep learning," "machine learning," "artificial intelligence," "neural net*," and "model."
- Restriction to the following journals: Radiology, Radiologia Medica, Investigative Radiology, and European Radiology.
- Limitation to publications from the year 2022.
- Final search strategy:

#	Searches
1	AI.mp. [mp=ti, ab, tx, ct, sh, bt, ot, nm, hw, fx, kf, ox, px, rx, an, ui, ds, on, sy, ux, mx]
2	deep learning.mp. [mp=ti, ab, tx, ct, sh, bt, ot, nm, hw, fx, kf, ox, px, rx, an, ui, ds, on, sy, ux, mx]
3	machine learning.mp. [mp=ti, ab, tx, ct, sh, bt, ot, nm, hw, fx, kf, ox, px, rx, an, ui, ds, on, sy, ux, mx]
4	artificial intelligence.mp. [mp=ti, ab, tx, ct, sh, bt, ot, nm, hw, fx, kf, ox, px, rx, an, ui, ds, on, sy, ux, mx]
5	neural net*.mp. [mp=ti, ab, tx, ct, sh, bt, ot, nm, hw, fx, kf, ox, px, rx, an, ui, ds, on, sy, ux, mx]
6	model.mp.
7	1 or 2 or 3 or 4 or 5 or 6
8	Radiology.m_jour
9	limit 8 to yr =”2022”
10	7 and 9
11	Radiologia Medica.m_jour
12	limit 11 to yr=”2022”
13	7 and 12
14	INVESTIGATIVE RADIOLOGY.m_jour
15	limit 14 to yr=”2022”
16	7 and 15
17	EUROPEAN RADIOLOGY.m_jour
18	limit 17 to yr=”2022”
19	7 and 18

The Embase search strategy included:

- Keywords related to artificial intelligence: "AI," "deep learning," "machine learning," "artificial intelligence," "neural net*," and "model."
- Restriction to the journal: Diagnostic and Interventional Imaging.
- Limitation to publications from the year 2022.
- Final search strategy:

#	Searches
1	‘ai
2	‘deep learning’
3	‘machine learning’
4	‘artificial intelligence’
5	‘neural net*’
6	‘model’
7	#1 OR #2 OR #3 OR #4 OR #5 OR #6
8	‘diagnostic and interventional imaging’/jt
9	#7 AND #8
10	#9 AND 2022:py

Supplementary Table 1: A LIST OF INCLUDED STUDIES

This table contains only a selection of items, and all raw data used for analysis can be accessed in spreadsheet form via the following link (https://drive.google.com/file/d/1ANH6I1OdgM_ifSaivGz8MsOvQqlMrOll/view?usp=sharing)

First Author	Journal Name	Model Purpose	Nationality of First Author	Image Modality	Image Body Part	Development Set Size (Total with Training/Tuning/Internal Test Split)	Model Type Category	Model Type	Code/Model Accessibility (for deep learning)	Model Availibility (for all model)
Zhong Y [1]	Radiology	Prognostic	China	Cross-Sectional	Thorax & Breast	2663 (2131/266/266)	DL	CNN [ResNet-152]	Yes	No
Zhang XQ [2]	Radiology	Diagnostic	China	Ultrasound	Abdomen	159 (No split provided)	Regression	Logistic regression	Not applicable	Yes
Zhang M [3]	Radiology	Diagnostic	USA	Cross-Sectional	Neuro & Head/Neck	263 (3:1 ratio, 10-fold cross validation)	DL	MLP [customized]	Yes	No
You S [4]	Radiology	Diagnostic	USA	Cross-Sectional	Thorax & Breast	139 (85/14/40)	DL	CNN [3D U-Net]	No	No
Wanders AJT [5]	Radiology	Prognostic	Netherlands	Projection-Based	Thorax & Breast	6883 (4819/2064, 10-fold cross validation)	DL	ANN [customized]	No	No
Verburg E [6]	Radiology	Diagnostic	Netherlands	Cross-Sectional	Thorax & Breast	4133 (8:2 ratio)	DL	CNN [VGG16 + VGG19]	Yes	Yes
Tallam H [7]	Radiology	Diagnostic	USA	Cross-Sectional	Abdomen	9463 (424/8/9031)	Regression	Logistic regression	Not applicable	Yes
Swinburne NC [8]	Radiology	Diagnostic	USA	Cross-Sectional	Neuro & Head/Neck	3816 (3716/100)	DL	Single-shot Detector [RetinaNet] + Two-shot Detector [Mask R-CNN]	No	No
Shoshan Y [9]	Radiology	Diagnostic	Israel	Projection-Based	Thorax & Breast	9919 (3948/1661/4310)	DL	CNN [InceptionResNetV2] + Single-shot Detector [RetinaNet]	Yes	No
Shin J [10]	Radiology	Prognostic	Korea	Cross-Sectional	Abdomen	898 (592/306, 10-fold cross validation)	Regression	Logistic regression with LASSO regularization	Not applicable	Yes
Sellergren AB [11]	Radiology	Diagnostic	USA	Projection-Based	Thorax & Breast	451549 (448019/3530)	DL	SupCon + CNN [EfficientNet-B7 + ResNet-101 + ResNet-152]	No	No
Ramtohul T [12]	Radiology	Prognostic	France	Cross-Sectional	Thorax & Breast	50 (No split provided)	Regression	Logistic regression	Not applicable	No
Pezel T [13]	Radiology	Prognostic	USA	Cross-Sectional	Thorax & Breast	1911 (No split provided)	Regression	Cox regression	Not applicable	No
Pease M [14]	Radiology	Prognostic	USA	Cross-Sectional	Neuro & Head/Neck	537 (70:10:20 ratio)	DL	CNN [AlexNet]	Yes	No
Nishii T [15]	Radiology	Diagnostic	Japan	Cross-Sectional	Thorax & Breast	180 (90/10/80)	DL	Residual Dense Network	Yes	No
Nam JG [16]	Radiology	Prognostic	Korea	Projection-Based	Thorax & Breast	4519 (3810/709)	DL	CNN [DenseNet-169]	Yes	No
Mu D [17]	Radiology	Diagnostic	China	Cross-Sectional	Thorax & Breast	605 (292/73/240)	DL	CNN [Customized]	Yes	No

Mohajer B [18]	Radiology	Prognostic	USA	Cross-Sectional	MSK	350 (segmentation task, 200/150), 1831 (prognostication task)	DL	CNN [2D U-Net]	Yes	No
Lee SE [19]	Radiology	Diagnostic	Korea	Multimodality	Thorax & Breast	477 (330/147)	Regression	Logistic regression	Not applicable	Yes
Lee SA [20]	Radiology	Diagnostic	Korea	Cross-Sectional	Thorax & Breast	245 (140/105)	Regression	Logistic regression	Not applicable	Yes
Kwon BR [21]	Radiology	Prognostic	Korea	Multimodality	Thorax & Breast	318 (Bootstrapping with 1000 resamples)	Regression	Cox regression	Not applicable	Yes
Krishnan AP [22]	Radiology	Prognostic	USA	Cross-Sectional	Neuro & Head/Neck	1574 (778/796, 3-fold cross validation)	DL	CNN [2.5D U-Net]	No	No
Jaramillo D [23]	Radiology	Prognostic	USA	Cross-Sectional	MSK	142 (10-fold cross validation)	Regression	Linear regression	Not applicable	Yes
Hou R [24]	Radiology	Diagnostic	USA	Projection-Based	Thorax & Breast	700 (400/300, 5-fold cross validation)	Regression	Logistic regression with L2 regularization	Not applicable	No
Fan M [25]	Radiology	Prognostic	China	Cross-Sectional	Thorax & Breast	265 (130/135, 10-fold cross validation)	Regression	Cox regression with elestic net regularization	Not applicable	Yes
Choe J [26]	Radiology	Diagnostic	Korea	Cross-Sectional	Thorax & Breast	N/A for training/validation, 80 for test	DL	CNN [Customized]	No	No
Chen Y [27]	Radiology	Diagnostic	China	Ultrasound	Neuro & Head/Neck	450 (5-fold cross validation)	DL	CNN [InceptionResNetV2]	Yes	No
Chen JV [28]	Radiology	Diagnostic	China	Cross-Sectional	Neuro & Head/Neck	469 (70:20:10 ratio)	DL	CNN [Customized]	Yes	No
Chen H [29]	Radiology	Diagnostic	China	Ultrasound	Abdomen	422 (337/85)	DL	CNN [ResNet-18 + ResNet-50]	Yes	No
Bressem KK [30]	Radiology	Diagnostic	Germany	Cross-Sectional	MSK	477 (404/73)	DL	CNN [U-Net + ResNet-101]	Yes	No
Benzakoun J [31]	Radiology	Diagnostic	France	Cross-Sectional	Neuro & Head/Neck	821 (1416 images, 1134/282)	DL	GAN [Ea-GAN]	Yes	Yes
Aquino GJ [32]	Radiology	Prognostic	Germany	Cross-Sectional	Thorax & Breast	196 (No split provided)	Regression	Cox regression	Not applicable	Yes
Alabed S [33]	Radiology	Prognostic	UK	Cross-Sectional	Thorax & Breast	4546 (539/4007)	DL	CNN [U-Net]	No	No
Yao F [34]	Radiologia Medica	Diagnostic	China	Cross-Sectional	Abdomen	173 (122/51)	Others	SVM with radial basis function kernel	Not applicable	No
Xue K [35]	Radiologia Medica	Diagnostic	China	Cross-Sectional	Abdomen	133 (92/41)	Regression	Logistic regression	Not applicable	Yes
Wang FH [36]	Radiologia Medica	Prognostic	China	Cross-Sectional	Abdomen	220 (131/89, 10-fold cross validation)	Regression	Cox regression with LASSO regularization	Not applicable	Yes
van der Lubbe M [37]	Radiologia Medica	Diagnostic	Netherlands	Cross-Sectional	Neuro & Head/Neck	260 (192/68 or 10-fold cross validation)	DL	MLP [customized]	No	No
Song W [38]	Radiologia Medica	Prognostic	China	Cross-Sectional	Abdomen	112 (No split provided)	Regression	Cox regression	Not applicable	Yes
Palmisano A [39]	Radiologia Medica	Prognostic	Italy	Cross-Sectional	Thorax & Breast	1125 (789/336, 10x5 repeated cross validation)	Others	9 machine learning algorithms	Not applicable	No
Han D [40]	Radiologia Medica	Prognostic	China	Cross-Sectional	Abdomen	132 (79/53, cross validation)	Regression	Cox regression with LASSO regularization	Not applicable	Yes

Gregucci F [41]	Radiologia Medica	Prognostic	Italy	Cross-Sectional	Abdomen	37 (No split provided)	Regression	Logistic regression	Not applicable	Yes
Granata V [42]	Radiologia Medica	Prognostic	Italy	Cross-Sectional	Abdomen	51 (10-fold cross validation)	Regression	Linear regression	Not applicable	Yes
Granata V [43]	Radiologia Medica	Diagnostic	Italy	Cross-Sectional	Abdomen	51 (10-fold cross validation)	Regression	Linear regression	Not applicable	Yes
Gitto S [44]	Radiologia Medica	Diagnostic	Italy	Cross-Sectional	MSK	101 (10-fold cross validation)	Others	SVM	Not applicable	No
Gao W [45]	Radiologia Medica	Prognostic	China	Cross-Sectional	Abdomen	472 (378/94)	DL	CNN [customized]	No	No
Fan Y [46]	Radiologia Medica	Prognostic	China	Cross-Sectional	Neuro & Head/Neck	230 (153/77, 10-fold cross validation)	Regression	Logistic regression with LASSO regularization	Not applicable	Yes
Cilla S [47]	Radiologia Medica	Diagnostic	Italy	Cross-Sectional	Thorax & Breast	30 (5-fold cross validation)	Regression	Logistic regression, SVM, and CART	Not applicable	Yes
Chiti G [48]	Radiologia Medica	Diagnostic	Italy	Cross-Sectional	Abdomen	78 (3:1 ratio)	Regression	LASSO regression	Not applicable	No
Chiloiro G [49]	Radiologia Medica	Diagnostic	Italy	Cross-Sectional	Abdomen	144 (No split provided)	Regression	Logistic regression	Not applicable	Yes
Caruso D [50]	Radiologia Medica	Prognostic	Italy	Cross-Sectional	Abdomen	112 (25/87, Synthetic test set)	Regression	Logistic regression	Not applicable	Yes
Calandrelli R [51]	Radiologia Medica	Prognostic	Italy	Cross-Sectional	Neuro & Head/Neck	105 (85/20, 3-fold cross validation)	Regression	Logistic regression	Not applicable	Yes
Autorino R [52]	Radiologia Medica	Prognostic	Italy	Cross-Sectional	Abdomen	142 (No split provided)	Regression	Logistic regression	Not applicable	Yes
Abdullah SS [53]	Radiologia Medica	Diagnostic	India	Projection-Based	MSK	1985 (1468/181/336)	DL	CNN [ResNet-50 + AlexNet] + Faster RCNN	No	No
Ziegelmayer S [54]	Investigative Radiology	Diagnostic	Germany	Cross-Sectional	Abdomen	60 (48/12, 4-fold cross validation)	DL	CNN [VGG19]	No	No
Wennmann M [55]	Investigative Radiology	Diagnostic	Germany	Cross-Sectional	MSK	81 (50/31)	Others	Random forest	Not applicable	No
Toda N [56]	Investigative Radiology	Diagnostic	Japan	Cross-Sectional	Abdomen	453 (10-fold cross validation)	DL	CNN [2D/3D U-Net]	No	No
Thomas MF [57]	Investigative Radiology	Diagnostic	Germany	Cross-Sectional	Neuro & Head/Neck	283 (231/12/40)	DL	GAN [pix2pix]	Yes	Yes
Storelli L [58]	Investigative Radiology	Prognostic	Italy	Cross-Sectional	Neuro & Head/Neck	325 (7:3 ratio)	DL	CNN [Customized]	No	No
Sexauer R [59]	Investigative Radiology	Diagnostic	Switzerland	Cross-Sectional	Thorax & Breast	655 (458/197)	DL	CNN [nnUNet]	Yes	Yes

Rudolph J [60]	Investigative Radiology	Diagnostic	Germany	Projection-Based	Thorax & Breast	Insufficient information	DL	CNN [Customized]	No	No
Lee SB [61]	Investigative Radiology	Diagnostic	Korea	Cross-Sectional	Abdomen	A single phantom study	DL	GAN [customized]	No	No
Huber FA [62]	Investigative Radiology	Diagnostic	Switzerland	Cross-Sectional	Multiple regions	46 (8/4/34)	DL	CNN [U-Net]	No	No
Haubold J [63]	Investigative Radiology	Diagnostic	Germany	Cross-Sectional	Abdomen	19 (16/3)	DL	CNN [CycleGAN]	No	No
Ammari S [64]	Investigative Radiology	Diagnostic	France	Cross-Sectional	Neuro & Head/Neck	145 (107/38, 5-fold cross validation)	DL	CNN [3D U-Net]	Yes	Yes
Afat S [65]	Investigative Radiology	Diagnostic	Germany	Cross-Sectional	Abdomen	Approximately 400	DL	CNN [Customized]	No	No
Zou M [66]	European Radiology	Diagnostic	China	Cross-Sectional	Neuro & Head/Neck	59 (No split provided)	Regression	Logistic regression	Not applicable	Yes
Zhu C [67]	European Radiology	Prognostic	China	Cross-Sectional	Abdomen	106 (73/33)	Regression	Logistic regression	Not applicable	Yes
Zhou Y [68]	European Radiology	Diagnostic	China	Cross-Sectional	Abdomen	216 (151/65, 10-fold cross validation)	Regression	Logistic regression	Not applicable	Yes
Zhou Y [69]	European Radiology	Prognostic	China	Cross-Sectional	Abdomen	191 (135/56, 10-fold cross validation)	Regression	Cox regression with LASSO regularization	Not applicable	Yes
Zhou JY [70]	European Radiology	Prognostic	China	Multimodality	Neuro & Head/Neck	119 (No split provided)	Regression	Logistic regression	Not applicable	No
Zhong J [71]	European Radiology	Prognostic	China	Cross-Sectional	MSK	144 (101/43, 5-fold cross validation)	Regression	Logistic regression and SVM	Not applicable	Yes
Zheng YM [72]	European Radiology	Diagnostic	China	Cross-Sectional	Neuro & Head/Neck	104 (10-fold cross validation)	Regression	Logistic regression with LASSO regularization	Not applicable	Yes
Zheng Y [73]	European Radiology	Diagnostic	China	Cross-Sectional	Neuro & Head/Neck	388 (272/116, 10-fold cross validation)	Others	SVM	Not applicable	No
Zheng H [74]	European Radiology	Diagnostic	USA	Cross-Sectional	Abdomen	244 (160/84, 5-fold cross validation)	Others	SVM	Not applicable	No
Zhao M [75]	European Radiology	Prognostic	China	Cross-Sectional	Abdomen	421 (80:20 MC cross validation)	Others	Bayesian Classifier, Multi-Gaussian Weighted Classified, Random Forest, and SVM	Not applicable	No
Zhang Q [76]	European Radiology	Prognostic	China	Cross-Sectional	Abdomen	79 (No split provided)	Regression	Cox regression	Not applicable	Yes
Zhang MZ [77]	European Radiology	Prognostic	China	Cross-Sectional	Neuro & Head/Neck	151 (77/74)	Others	Extra trees	Not applicable	No

Zhang K [78]	European Radiology	Diagnostic	China	Cross-Sectional	Abdomen	129 (No split provided)	Regression	Logistic regression	Not applicable	Yes
Zhang J [79]	European Radiology	Prognostic	China	Cross-Sectional	Thorax & Breast	160 (112/48)	Regression	Logistic regression	Not applicable	Yes
Zhang H [80]	European Radiology	Prognostic	China	Cross-Sectional	Neuro & Head/Neck	212 (148/64, bootstrapping with 1000 resamples)	Regression	Cox regression	Not applicable	Yes
Zhang H [81]	European Radiology	Diagnostic	China	Cross-Sectional	Abdomen	138 (103/35, 10-fold cross validation)	Regression	Logistic regression	Not applicable	Yes
Zhang G [82]	European Radiology	Diagnostic	China	Cross-Sectional	Abdomen	366 (293/73, 10-fold cross validation)	Regression	Logistic regression	Not applicable	Yes
Yu Y [83]	European Radiology	Prognostic	China	Cross-Sectional	Abdomen	182 (128/54, 5-fold cross validation)	Regression	Decision tree, Random forest, SVM, and logistic regression	Not applicable	No
Yoo HJ [84]	European Radiology	Diagnostic	Korea	Projection-Based	MSK	100 (bootstrapping with 1000 resamples)	Regression	General linear model	Not applicable	Yes
Yang X [85]	European Radiology	Diagnostic	China	Cross-Sectional	Thorax & Breast	408 (327/81, 5-fold cross validation)	Regression	Logistic regression	Not applicable	No
Yang SS [86]	European Radiology	Prognostic	China	Multimodality	Neuro & Head/Neck	895 (542/353)	Regression	Cox regression	Not applicable	Yes
Yan C [87]	European Radiology	Diagnostic	China	Cross-Sectional	Thorax & Breast	527 (No split provided)	DL	CNN [SeNet-ResNet-18]	No	No
Xu Z [88]	European Radiology	Diagnostic	China	Cross-Sectional	Thorax & Breast	677 (No split provided)	Regression	Logistic regression	Not applicable	No
Xu Y [89]	European Radiology	Diagnostic	China	Cross-Sectional	Thorax & Breast	696 (5-fold cross validation)	Others	Random forest	Not applicable	No
Xu XQ [90]	European Radiology	Prognostic	China	Cross-Sectional	Neuro & Head/Neck	84 (No split provided)	Regression	Logistic regression	Not applicable	No
Xu H [91]	European Radiology	Diagnostic	China	Cross-Sectional	Thorax & Breast	294 (183/111)	Regression	Logistic regression	Not applicable	Yes
Xie X [92]	European Radiology	Diagnostic	China	Cross-Sectional	Neuro & Head/Neck	89 (71/18, 5-fold cross validation)	DL	CNN [Customized]	No	No
Xie T [93]	European Radiology	Diagnostic	China	Cross-Sectional	Thorax & Breast	99 (No split provided)	Regression	Logistic regression	Not applicable	No
Wu S [94]	European Radiology	Diagnostic	China	Cross-Sectional	Neuro & Head/Neck	111 (78/33, 10-fold cross validation)	Regression	Logistic regression	Not applicable	Yes
Wu S [95]	European Radiology	Diagnostic	China	Ultrasound	Abdomen	304 (208/96, 5-fold cross valdiation)	DL	CNN [InceptionV3 + ResNet-50 + Xception]	No	No
Wu K [96]	European Radiology	Prognostic	China	Cross-Sectional	Abdomen	443 (351/88)	Others	PCA + SVD + XGBoost	Not applicable	No

Wu C [97]	European Radiology	Diagnostic	China	Projection-Based	MSK	845 (788/57)	DL	CNN [HRNet]	No	No
Wood DA [98]	European Radiology	Diagnostic	UK	Cross-Sectional	Neuro & Head/Neck	126526 (124277/899/1350)	DL	LLM [BioBERT]	No	No
Wesp P [99]	European Radiology	Diagnostic	Germany	Cross-Sectional	Abdomen	63 (No split provided)	DL	CNN [Customized]	Yes	Yes
Wang Y [100]	European Radiology	Prognostic	China	Cross-Sectional	Abdomen	131 (No split provided)	Regression	Cox regression	Not applicable	Yes
Wang Y [101]	European Radiology	Prognostic	China	Cross-Sectional	Neuro & Head/Neck	184 (5-fold cross validation)	Others	SVM	Not applicable	No
Wang S [102]	European Radiology	Diagnostic	China	Projection-Based	Thorax & Breast	190 (1:1 ratio, 10-fold cross validation)	Regression	LASSO regression	Not applicable	Yes
Wang R [103]	European Radiology	Prognostic	China/USA	Cross-Sectional	Thorax & Breast	1051 (737/105/209)	DL	CNN [3D U-Net + EfficientNet-B4]	Yes	Yes
Wang R [104]	European Radiology	Prognostic	China	Cross-Sectional	Thorax & Breast	201 (5-fold cross validation)	Regression	Logistic regression	Not applicable	Yes
Wang Q [105]	European Radiology	Diagnostic	China	Ultrasound	Thorax & Breast	743 (769 lesions, 500/100/169)	DL	CNN [ResNet networks]	No	No
Wang L [106]	European Radiology	Diagnostic	China	Cross-Sectional	Thorax & Breast	903 (965 lesions, 482/121/362)	DL	CNN [ResNet-50]	No	No
Wang J [107]	European Radiology	Diagnostic	China	Ultrasound	Neuro & Head/Neck	2992 (2794/198)	DL	CNN [ResNet-50]	Yes	No
Wang J [108]	European Radiology	Prognostic	China	Multimodality	Neuro & Head/Neck	61 (7:3 ratio)	Regression	Logistic regression	Not applicable	No
Wang H [109]	European Radiology	Prognostic	China	Cross-Sectional	Abdomen	296 (No split provided)	Regression	Logistic regression	Not applicable	Yes
Wackenthaler A [110]	European Radiology	Prognostic	France	Cross-Sectional	Abdomen	82 (No split provided)	Regression	Cox regression	Not applicable	Yes
von Schacky CE [111]	European Radiology	Diagnostic	Germany	Projection-Based	MSK	880 (614/133/133)	DL	ANN [customized]	No	No
Visser JJ [112]	European Radiology	Diagnostic	Netherlands	Multimodality	Multiple regions	2306 reports (1306/1000)	Others	Random forest	Not applicable	No
Uhlig J [113]	European Radiology	Diagnostic	Germany/USA	Multimodality	Abdomen	170 (10-fold cross validation)	Others	Random forest	Not applicable	No
Tzanis E [114]	European Radiology	Prognostic	Greece	Cross-Sectional	Neuro & Head/Neck	343 (231/112)	DL	DNN [Customized]	Yes	Yes
Tsuchiya M [115]	European Radiology	Diagnostic	Japan	Cross-Sectional	Thorax & Breast	88 (augmented 300 lesion: 200/100, 10-fold cross validation)	Others	SVM	Not applicable	No

Tran A [116]	European Radiology	Diagnostic	France	Cross-Sectional	MSK	17738 (19765 images, 13836/3953/1976)	DL	CNN [Customized]	No	No
Tomita H [117]	European Radiology	Prognostic	Japan	Cross-Sectional	Neuro & Head/Neck	70 (49/21)	DL	CNN [Xception]	No	No
Ternifi R [118]	European Radiology	Diagnostic	USA	Ultrasound	Thorax & Breast	521 (No split provided)	Regression	Logistic regression	Not applicable	No
Tang WJ [119]	European Radiology	Diagnostic	China	Cross-Sectional	Thorax & Breast	133 (92/41, 5-fold cross validation)	Regression	Logistic regression	Not applicable	No
Tang CX [120]	European Radiology	Prognostic	China	Cross-Sectional	Thorax & Breast	466 (No split provided)	Others	Flow-chart scoring using CT_FFR	Not applicable	Yes
Sushentsev N [121]	European Radiology	Prognostic	UK	Cross-Sectional	Abdomen	64 (leave-one-out cross-validation)	Regression	Parenclitic network, LASSO regression, random forest	Not applicable	No
Sun X [122]	European Radiology	Diagnostic	China	Cross-Sectional	Neuro & Head/Neck	358 (5-fold cross validation)	DL	CNN [ResNet-34]	No	No
Sun SH [123]	European Radiology	Prognostic	USA	Cross-Sectional	Abdomen	206 (138/68)	Others	Random forest based scoring system	Not applicable	Yes
Sun J [124]	European Radiology	Diagnostic	China	Cross-Sectional	Thorax & Breast	1393 (837/278/278)	DL	CNN [ResNet-18 + ResNet-50 + U-Net] + Attention mechanism-based classifier	No	No
Song SE [125]	European Radiology	Diagnostic	Korea	Cross-Sectional	Thorax & Breast	300 (10-fold cross validation)	Regression	Logistic regression	Not applicable	Yes
Skarping I [126]	European Radiology	Prognostic	Sweden	Projection-Based	Thorax & Breast	453 (400/53)	DL	CNN [ResNet-18] + Transformer	No	No
Sieren MM [127]	European Radiology	Diagnostic	Germany	Cross-Sectional	Thorax & Breast	191 (135/19/37)	DL	CNN [3D U-Net]	No	No
Si N [128]	European Radiology	Diagnostic	China	Cross-Sectional	Thorax & Breast	210 (147/63, 5-fold cross validation)	Regression	Logistic regression	Not applicable	Yes
Shu Z [129]	European Radiology	Diagnostic	China	Cross-Sectional	Abdomen	317 (221/96)	Regression	Logistic regression	Not applicable	Yes
Sheng R [130]	European Radiology	Prognostic	China	Cross-Sectional	Abdomen	61 (No split provided)	Regression	Cox regression	Not applicable	Yes
Sheng DL [131]	European Radiology	Prognostic	China	Multimodality	Thorax & Breast	636 (446/190)	Regression	Cox regression	Not applicable	Yes
Rui W [132]	European Radiology	Diagnostic	China	Cross-Sectional	Neuro & Head/Neck	302 (242/60, 5-fold cross validation)	DL	ANN [customized]	No	No
Ruhling S [133]	European Radiology	Diagnostic	Germany	Cross-Sectional	MSK	193 (154/39, 3-fold cross validation)	DL	CNN [DenseNet]	Yes	No
Rouviere O [134]	European Radiology	Diagnostic	France	Cross-Sectional	Abdomen	211 (130/81)	DL	CNN [Customized]	No	No

Ren J [135]	European Radiology	Diagnostic	China	Cross-Sectional	Neuro & Head/Neck	55 (No split provided)	Regression	Logistic regression	Not applicable	No
Pontillo G [136]	European Radiology	Prognostic	Italy	Cross-Sectional	Neuro & Head/Neck	653 (10-fold cross validation)	Others	SuStaIn algorithm	Not applicable	No
Pfob A [137]	European Radiology	Diagnostic	Germany/USA	Ultrasound	Thorax & Breast	915 (10-fold cross validation)	Regression	Logistic regression with elastic net regularization	Not applicable	Yes
Peng WL [138]	European Radiology	Diagnostic	China	Cross-Sectional	Thorax & Breast	135 (94/41, 5-fold cross validation)	Others	Tree-based pipeline optimization tool	Not applicable	No
Park YW [139]	European Radiology	Prognostic	Korea	Cross-Sectional	Neuro & Head/Neck	61 (bootstrapping with 1000 resamples)	Regression	Cox regression with Elestic net regularization	Not applicable	Yes
Park YW [140]	European Radiology	Diagnostic	Korea	Cross-Sectional	Neuro & Head/Neck	302 (211/91, 10-fold cross validation)	Others	Light gradient boosting model	Not applicable	No
Park S [141]	European Radiology	Diagnostic	Korea	Cross-Sectional	Thorax & Breast	576 (384/182)	Regression	Logistic regression	Not applicable	Yes
Park HY [142]	European Radiology	Prognostic	Korea	Cross-Sectional	Neuro & Head/Neck	60 (bootstrapping with 1000 resamples)	Regression	Cox regression	Not applicable	Yes
Park D [143]	European Radiology	Prognostic	Korea	Cross-Sectional	Thorax & Breast	106 (5-fold cross validation)	Others	Random forest	Not applicable	No
Ou C [144]	European Radiology	Prognostic	China/Australia	Cross-Sectional	Neuro & Head/Neck	93 (5-fold cross validation)	DL	CNN [ResNet-18]	Yes	No
Nowak S [145]	European Radiology	Diagnostic	Germany	Cross-Sectional	MSK	1466 (1164/302)	DL	CNN [nnU-Net + CDFNet]	No	No
Noortman WA[146]	European Radiology	Diagnostic	Netherlands	Cross-Sectional	Abdomen	38 (5-fold cross validation)	Regression	Logistic regression	Not applicable	No
Noguchi S [147]	European Radiology	Diagnostic	Japan	Cross-Sectional	MSK	732 (632/40/60)	DL	CNN [2D U-Net + 3D ResNet]	Yes	No
Nagaraj Y [148]	European Radiology	Diagnostic	Netherlands	Cross-Sectional	Thorax & Breast	658 (multiple data scenarios)	DL	MLP [customized]	No	No
Muller L [149]	European Radiology	Prognostic	Germany	Cross-Sectional	Abdomen	327 (70/30/227)	DL	CNN [3D U-Net]	No	No
Meng Y [150]	European Radiology	Diagnostic	China	Projection-Based	Thorax & Breast	1125 images (820/205/100)	DL	CNN [U-Net]	No	No
Meng J [151]	European Radiology	Diagnostic	China	Cross-Sectional	Abdomen	179 (114/65, 10-fold cross validation)	DL	CNN [ResNet]	No	No
Matsumoto T [152]	European Radiology	Diagnostic	Japan	Projection-Based	Thorax & Breast	7047 (5637/704/706)	DL	CNN [EfficientNet]	Yes	No
Mao N [153]	European Radiology	Prognostic	China	Projection-Based	Thorax & Breast	118 (81/37, 10-fold cross validation)	Regression	Logistic regression with LASSO regularization	Not applicable	Yes

Ma Y [154]	European Radiology	Diagnostic	China	Cross-Sectional	Abdomen	201 (No split provided)	Regression	Logistic regression	Not applicable	Yes
Ma Q [155]	European Radiology	Diagnostic	China	Cross-Sectional	Neuro & Head/Neck	317 (5-fold cross validation)	Regression	SVM + logistic regression	Not applicable	Yes
Ma M [156]	European Radiology	Diagnostic	China	Multimodality	Thorax & Breast	600 (450/150, 5-fold cross validation)	Regression	5 machine learning algorithms (with logistic regression)	Not applicable	No
Luo X [157]	European Radiology	Diagnostic	China	Cross-Sectional	Neuro & Head/Neck	112 (83/29)	Others	Linear SVM	Not applicable	Yes
Luo S [158]	European Radiology	Diagnostic	China	Cross-Sectional	Abdomen	177 (5-fold cross validation)	Regression	8 Classificiation algorithms (with logistic regression)	Not applicable	No
Luo N [159]	European Radiology	Prognostic	China	Cross-Sectional	Abdomen	502 (No split provided)	Regression	Logistic regression	Not applicable	Yes
Ludemann W [160]	European Radiology	Prognostic	Germany	Cross-Sectional	Abdomen	58 (No split provided)	Regression	Logistic regression	Not applicable	Yes
Lu SS [161]	European Radiology	Prognostic	China	Cross-Sectional	Neuro & Head/Neck	112 (No split provided)	Regression	Logistic regression	Not applicable	No
Lopes RR [162]	European Radiology	Prognostic	Netherlands	Cross-Sectional	Thorax & Breast	1364 (5-fold and 10-fold cross validation)	Regression	Logistic regression	Not applicable	Yes
Liu Y [163]	European Radiology	Prognostic	China	Ultrasound	Neuro & Head/Neck	148 (104/44)	Regression	Logistic regression	Not applicable	Yes
Liu X [164]	European Radiology	Prognostic	China	Cross-Sectional	Neuro & Head/Neck	112 (78/34, leave-one-out cross validation)	Regression	Logistic regression with LASSO regularization	Not applicable	Yes
Liu S [165]	European Radiology	Prognostic	China	Cross-Sectional	MSK	151 (No split provided)	DL	CNN [ResNet-34]	No	No
Liu R [166]	European Radiology	Diagnostic	China	Projection-Based	MSK	643 (982 images, 784/97/101)	DL	CNN [Inception-V3]	No	No
Liu K [167]	European Radiology	Prognostic	China	Cross-Sectional	Thorax & Breast	204 (142/62)	DL	CNN [VGG19]	No	No
Liu J [168]	European Radiology	Diagnostic	China	Cross-Sectional	MSK	241 (5-fold cross validation)	Regression	Logistic regression	Not applicable	No
Liu B [169]	European Radiology	Diagnostic	China	Cross-Sectional	Abdomen	114 (74/40)	DL	CNN [LeNet-5]	No	No
Lin X [170]	European Radiology	Prognostic	China	Cross-Sectional	Neuro & Head/Neck	90 (74/16, 10-fold cross validation)	Regression	Linear mixed model	Not applicable	Yes
Lin FY [171]	European Radiology	Diagnostic	China	Cross-Sectional	Thorax & Breast	888 (No split provided)	Others	Random forest	Not applicable	No
Lim RP [172]	European Radiology	Diagnostic	Australia	Cross-Sectional	Thorax & Breast	454 (Complicated setting)	DL	CNN [Customized]	Yes	Yes

Liang H [173]	European Radiology	Prognostic	China	Cross-Sectional	Thorax & Breast	2265 (Multiple experimental setting)	DL	CNN [ResNet-18 + ResNeXt-50 + DenseNet-121] + DeepLabV3 + RNN	No	No
Li ZC [174]	European Radiology	Prognostic	China	Cross-Sectional	Neuro & Head/Neck	1091 (935/156/0)	DL	CNN [Attention-based CNN with ResNet-34 backbone + original ResNet-34]	Yes	Yes
Li Y [175]	European Radiology	Prognostic	China	Cross-Sectional	Neuro & Head/Neck	352 (No split provided)	Regression	Logistic regression	Not applicable	No
Li Y [176]	European Radiology	Diagnostic	China	Cross-Sectional	Thorax & Breast	443 (5-fold cross validation)	DL	CNN [U-Net + 3DNet]	No	No
Li Y [177]	European Radiology	Diagnostic	China	Cross-Sectional	Neuro & Head/Neck	1016 (780/236, 5-fold cross validation)	DL	CNN [ResNet-18]	No	No
Li Y [178]	European Radiology	Diagnostic	China	Cross-Sectional	Abdomen	235 (194/41)	Regression	Logistic regression	Not applicable	No
Li Y [179]	European Radiology	Prognostic	China	Cross-Sectional	Thorax & Breast	108 (87/21, bootstrapping with 1000 resamples)	Regression	Logistic regression	Not applicable	Yes
Li XN [180]	European Radiology	Diagnostic	China	Cross-Sectional	Thorax & Breast	44 (36/8. 5-fold cross validation)	DL	MLP [customized]	No	No
Li X [181]	European Radiology	Prognostic	China	Cross-Sectional	Abdomen	1116 (892/244)	DL	CNN [DenseNet-121]	No	No
Li M [182]	European Radiology	Diagnostic	China	Cross-Sectional	Neuro & Head/Neck	585 (390/195)	Others	Random forest	Not applicable	Yes
Lee SB [183]	European Radiology	Diagnostic	Korea	Cross-Sectional	Multiple regions	26 (31 scans, 27/2/2)	DL	CNN [3D U-Net]	No	No
Lee JY [184]	European Radiology	Diagnostic	Korea	Cross-Sectional	Thorax & Breast	288 (291 lesions: 218/73)	DL	ANN [customized]	No	No
Lee JE [185]	European Radiology	Diagnostic	Korea	Cross-Sectional	Abdomen	152 (bootstrapping with 500 resamples)	Regression	Logistic regression	Not applicable	Yes
Laredo C [186]	European Radiology	Prognostic	Spain	Cross-Sectional	Neuro & Head/Neck	828 (654/174)	Regression	Logistic regression with advanced analysis of interaction	Not applicable	Yes
Kruger J [187]	European Radiology	Diagnostic	Germany	Cross-Sectional	Neuro & Head/Neck	1809 (No split provided)	DL	CNN [U-Net]	No	No
Koo CW [188]	European Radiology	Diagnostic	USA	Cross-Sectional	Thorax & Breast	1085 (911/174, bootstrapping with 5000 resamples)	Others	XGBoost	Not applicable	No
Kolossvary M [189]	European Radiology	Prognostic	USA	Cross-Sectional	Thorax & Breast	69 (No split provided)	Regression	Linear mixed model	Not applicable	No
Klontzas ME [190]	European Radiology	Prognostic	Greece	Cross-Sectional	MSK	257 (507 images, 80% training, 20% external validation, leaving out one-third validation)	Others	Random forest	Not applicable	No

Kimura K [191]	European Radiology	Prognostic	Japan	Cross-Sectional	Abdomen	45 (10-fold cross validation)	Others	RF and SVM	Not applicable	No
Kim E [192]	European Radiology	Prognostic	Korea	Cross-Sectional	Thorax & Breast	298 (5-fold cross validation)	Regression	Cox regression with LASSO regularization	Not applicable	Yes
Kim C [193]	European Radiology	Diagnostic	Korea	Projection-Based	Thorax & Breast	2017 (1614/403)	DL	CNN [U-Net + R-CNN]	No	No
Kikuchi Y [194]	European Radiology	Diagnostic	Japan	Cross-Sectional	Neuro & Head/Neck	84 (50/34)	DL	CNN [DeepMedic network]	No	No
Kapsner LA [195]	European Radiology	Diagnostic	Germany	Cross-Sectional	Thorax & Breast	1794 (1378/416, 5-fold cross validation)	DL	CNN [DenseNet-121 + ResNet-18]	No	No
Kang JJ [196]	European Radiology	Diagnostic	China	Cross-Sectional	Neuro & Head/Neck	159 (7:3 ratio)	Others	SVM	Not applicable	No
Jung W [197]	European Radiology	Diagnostic	Korea	Cross-Sectional	Neuro & Head/Neck	342 (146/14/182)	DL	DNN [Customized]	No	No
Juan CJ [198]	European Radiology	Diagnostic	Taiwan	Cross-Sectional	Neuro & Head/Neck	188 (121/67)	DL	CNN [U-Net]	No	No
Jonske F [199]	European Radiology	Diagnostic	Germany	Multimodality	Multiple regions	11934 imaging series from 4597 separate examination	DL	CNN [ResNet-152 + DenseNet-161]	Yes	Yes
Jing X [200]	European Radiology	Diagnostic	Netherlands	Cross-Sectional	Thorax & Breast	488 (339/149)	DL	CNN [3D U-Net + ResNet-34]	No	No
Jin X [201]	European Radiology	Diagnostic	China	Cross-Sectional	Thorax & Breast	404 (312/92/0)	DL	CNN [Mask-RCNN]	No	No
Jiang YW [202]	European Radiology	Diagnostic	China	Cross-Sectional	MSK	386 (270/116, 10-fold cross validation)	Regression	Logistic regression with LASSO regularization	Not applicable	Yes
Jiang M [203]	European Radiology	Diagnostic	China	Ultrasound	Thorax & Breast	303 (10-fold cross validation)	Regression	Logistic regression	Not applicable	Yes
Jiang L [204]	European Radiology	Diagnostic	China	Cross-Sectional	Neuro & Head/Neck	410 (nested cross validation)	DL	ANN [customized]	No	No
Jiang C [205]	European Radiology	Prognostic	China	Cross-Sectional	Abdomen	140 (100/40, 10-fold cross validation)	Regression	Cox regression	Not applicable	No
Jiang C [206]	European Radiology	Prognostic	China	Cross-Sectional	Multiple regions	297 (5-fold cross validation)	DL	CNN [nnUNet]	Yes	Yes
Jayaprakasam VS [207]	European Radiology	Prognostic	USA	Cross-Sectional	Abdomen	236 (5-fold cross validation)	Regression	SVM with Elestic net regularization	Not applicable	No
Javorszky N [208]	European Radiology	Diagnostic	Hungary	Cross-Sectional	Thorax & Breast	308 (186/61/61)	DL	CNN [U-Net]	Yes	Yes
Jang EB [209]	European Radiology	Prognostic	Korea	Multimodality	Neuro & Head/Neck	220 (10-fold cross validation)	Others	Imaging-based scoring system	Not applicable	Yes

Huang LT [210]	European Radiology	Diagnostic	Taiwan	Cross-Sectional	Thorax & Breast	428 (130/298, 5-fold cross validation)	DL	CNN [Attention U-Net + ResNeXt]	No	No
Huang L [211]	European Radiology	Diagnostic	China	Cross-Sectional	Thorax & Breast	203 (149/54)	Regression	Logistic regression	Not applicable	Yes
Huang J [212]	European Radiology	Diagnostic	China	Ultrasound	Abdomen	155 (No split provided)	Regression	Logistic regression	Not applicable	Yes
Huang J [213]	European Radiology	Diagnostic	China	Ultrasound	Abdomen	104 (80/24, 5-fold cross validation)	DL	CNN [ResNeXt-50]	No	No
Hou J [214]	European Radiology	Prognostic	China	Cross-Sectional	Neuro & Head/Neck	406 (285/121, 10-fold cross validation)	Regression	Logistic regression	Not applicable	Yes
Hosseinzadeh M [215]	European Radiology	Diagnostic	Netherlands	Cross-Sectional	Abdomen	2438 (1586/366/486)	DL	CNN [U-Net]	No	No
Hinzpeter R [216]	European Radiology	Diagnostic	Switzerland	Cross-Sectional	MSK	67 (augmented 410 lesion, 328/82, Leave-one-out cross-validation)	Others	Random forest	Not applicable	No
Hejduk P [217]	European Radiology	Diagnostic	Switzerland	Ultrasound	Thorax & Breast	113 (645 images, 367/150/128)	DL	CNN [Customized]	No	No
Healy GM [218]	European Radiology	Prognostic	Canada	Cross-Sectional	Abdomen	352 (5-fold cross validation)	Regression	Cox regression with LASSO regularization	Not applicable	Yes
He Z [219]	European Radiology	Diagnostic	China	Cross-Sectional	Neuro & Head/Neck	298 (209/89, 10-fold cross validation)	Others	XGBoost, SVM, DT	Not applicable	No
Han S [220]	European Radiology	Prognostic	Korea	Cross-Sectional	Thorax & Breast	106 (No split provided)	Regression	Cox regression	Not applicable	Yes
Guo X [221]	European Radiology	Diagnostic	China	Cross-Sectional	Neuro & Head/Neck	347 (226/76/45)	DL	CNN [ResNet-18]	No	No
Gu J [222]	European Radiology	Prognostic	China	Ultrasound	Thorax & Breast	168 (126/42, 5-fold cross validation)	DL	CNN [DenseNet-121]	No	No
Giannakopoulos P [223]	European Radiology	Prognostic	Switzerland	Cross-Sectional	Neuro & Head/Neck	80 (5-fold cross validation)	Regression	Logistic regression	Not applicable	Yes
Ghosh A [224]	European Radiology	Diagnostic	USA	Cross-Sectional	Multiple regions	62 (No split provided)	Regression	Logistic regression	Not applicable	Yes
Gerson R [225]	European Radiology	Diagnostic	Canada	Cross-Sectional	Abdomen	52 (No split provided)	Regression	Logistic regression	Not applicable	No
Garrido-Oliver J [226]	European Radiology	Diagnostic	Spain	Cross-Sectional	Thorax & Breast	404 (323/81)	DL	CNN [3D nnU-Net] + DQN	No	No
Frood R [227]	European Radiology	Prognostic	UK	Cross-Sectional	Multiple regions	289 (231/58, 5-fold cross validation)	Regression	Logistic regression	Not applicable	Yes
Fan Y [228]	European Radiology	Diagnostic	China	Cross-Sectional	MSK	160 (106/54, 10-fold cross validation)	Regression	Logistic regression	Not applicable	Yes

Eifer M [229]	European Radiology	Diagnostic	Israel	Cross-Sectional	Thorax & Breast	99 (165 lesion, 132/33, 5-fold cross validation)	Others	Random forest and KNN	Not applicable	No
Duan C [230]	European Radiology	Diagnostic	China	Cross-Sectional	Neuro & Head/Neck	117 (70/12/35)	DL	CNN [customized]	No	No
Duan C [231]	European Radiology	Diagnostic	China	Cross-Sectional	Thorax & Breast	106 (101/5)	DL	DC-RDN	No	No
Du S [232]	European Radiology	Prognostic	China	Cross-Sectional	Thorax & Breast	137 (102/35, bootstrapping with 1000 resamples)	Regression	Logistic regression	Not applicable	Yes
Dot G [233]	European Radiology	Diagnostic	France	Cross-Sectional	Neuro & Head/Neck	453 (300/153, 5-fold cross validation)	DL	CNN [nnU-Net]	No	No
Dong Y [234]	European Radiology	Prognostic	China	Cross-Sectional	Thorax & Breast	192 (128/64, 5-fold cross validation)	Regression	Logistic regression	Not applicable	Yes
Dong SY [235]	European Radiology	Prognostic	China	Cross-Sectional	Abdomen	214 (169/45)	Regression	Cox regression	Not applicable	Yes
Dong C [236]	European Radiology	Diagnostic	China	Cross-Sectional	Neuro & Head/Neck	94 (10-fold cross validation)	Regression	Logistic regression	Not applicable	Yes
Dominique C [237]	European Radiology	Diagnostic	France	Projection-Based	Thorax & Breast	389 (2460 images, 1574/390/496)	DL	CNN [DenseNet-121]	No	No
Demirjian NL [238]	European Radiology	Diagnostic	USA	Cross-Sectional	Abdomen	397 (5-fold cross validation)	Regression	Random forest, Adaboost, and Elastic Net	Not applicable	No
Demircioglu A [239]	European Radiology	Diagnostic	Germany	Projection-Based	MSK	2677 (2350/327, 5-fold cross validation)	DL	CNN [ResNet-34]	No	No
Dana J [240]	European Radiology	Diagnostic	France/Canada	Cross-Sectional	Abdomen	149 (10-fold cross validation)	Regression	Logistic regression with L1 regularization	Not applicable	Yes
Dai Q [241]	European Radiology	Diagnostic	China	Ultrasound	Neuro & Head/Neck	822 (575/247)	Regression	Logistic regression	Not applicable	Yes
Dai M [242]	European Radiology	Diagnostic	China	Cross-Sectional	Abdomen	172 (7:3 ratio)	DL	CNN [ResNet-50]	No	No
Corrado PA [243]	European Radiology	Diagnostic	USA	Cross-Sectional	Thorax & Breast	106 (80/26)	DL	Fully convolutional network	No	No
Chu F [244]	European Radiology	Prognostic	China	Cross-Sectional	Abdomen	434 (303/131, 10-fold cross validation)	Regression	Cox regression with LASSO regularization	Not applicable	Yes
Cheng J [245]	European Radiology	Prognostic	China	Projection-Based	Thorax & Breast	546 (8:2)	DL	CNN [ResNet-50] + Vision Transformer	No	No
Cheng B [246]	European Radiology	Diagnostic	China	Cross-Sectional	Thorax & Breast	564 (464/100, 5-fold cross validation)	Others	Gradient boosting decision tree	Not applicable	No
Chen ZW [247]	European Radiology	Diagnostic	China	Cross-Sectional	Thorax & Breast	659 (462/197)	Regression	5 machine learning algorithms (with logistic regression)	Not applicable	No

Chen W [248]	European Radiology	Prognostic	China	Projection-Based	MSK	1099 (824/275, 5-fold cross validation)	DL	CNN [ResNeSt-50]	Yes	No
Chen M [249]	European Radiology	Prognostic	China	Cross-Sectional	Abdomen	172 (132/40, 10-fold cross validation)	DL	DNN [custmomized]	No	No
Chen J [250]	European Radiology	Prognostic	China	Cross-Sectional	Neuro & Head/Neck	136 (95/41, 8-fold cross validation)	Regression	Cox regression	Not applicable	Yes
Chen H [251]	European Radiology	Diagnostic	China	Cross-Sectional	Neuro & Head/Neck	110 (71/39, leave-one-out cross valiation)	Regression	SVM + Multiple linear regression	Not applicable	No
Chen H [252]	European Radiology	Diagnostic	China	Cross-Sectional	Neuro & Head/Neck	545 (307/238)	DL	CNN [Modified attention U-Net]	Yes	No
Chen C [253]	European Radiology	Diagnostic	China	Cross-Sectional	Neuro & Head/Neck	152 (7:3 ratio, leave-one-out CV)	Regression	10 classification algorithms (with logistic regression)	Not applicable	No
Cha DI [254]	European Radiology	Diagnostic	Korea	Cross-Sectional	Abdomen	451 (bootstrapping with 1000 resamples)	Regression	Logistic regression	Not applicable	Yes
Cayot B [255]	European Radiology	Diagnostic	France	Cross-Sectional	Abdomen	88 (64/24)	DL	CNN [3D U-Net]	No	No
Cai S [256]	European Radiology	Diagnostic	China	Cross-Sectional	Thorax & Breast	520 (416/104, 10-fold cross validation)	Others	Random Forest and SVM	Not applicable	No
Brandt V [257]	European Radiology	Diagnostic	USA	Cross-Sectional	Thorax & Breast	95 (95/0/0)	Others	Logic-based decision based on CT-FFR	Not applicable	Yes
Brandt V [258]	European Radiology	Diagnostic	USA/Germany	Cross-Sectional	Thorax & Breast	128 (No split provided)	Regression	Logistic regression	Not applicable	No
Bleker J [259]	European Radiology	Diagnostic	Netherlands	Cross-Sectional	Abdomen	427 (524 lesions, 419/105, 10-fold cross validation)	DL	CNN [3D U-Net]	No	No
Bao D [260]	European Radiology	Prognostic	China	Cross-Sectional	Neuro & Head/Neck	216 (136/80)	Regression	Logistic regression	Not applicable	Yes
Badic B [261]	European Radiology	Prognostic	France	Cross-Sectional	Abdomen	193 (136/57)	Regression	Logistic regression, Random forest, SVM	Not applicable	No
Aquino GJ [262]	European Radiology	Diagnostic	USA	Cross-Sectional	Thorax & Breast	555 (443/57/55)	DL	I2I + cVAE	No	No
Annovazzi A [263]	European Radiology	Prognostic	Italy	Cross-Sectional	Multiple regions	57 (No split provided)	Regression	Cox regression	Not applicable	Yes
Tao J [264]	Diagnostic and Interventional Imaging	Diagnostic	China	Cross-Sectional	Thorax & Breast	203 (153/50, 5-fold cross validation)	DL	CNN [customized]	No	No
Ren T [265]	Diagnostic and Interventional Imaging	Diagnostic	China	Cross-Sectional	Abdomen	121 (No split provided)	Others	N/A	Not applicable	No

Paul JF [266]	Diagnostic and Interventional Imaging	Diagnostic	France	Cross-Sectional	Thorax & Breast	453 (400/53)	DL	CNN [InceptionV3]	No	No
Humbert C [267]	Diagnostic and Interventional Imaging	Prognostic	France	Cross-Sectional	Abdomen	95 (No split provided)	Regression	Logistic regression	Not applicable	Yes
Fabry V [268]	Diagnostic and Interventional Imaging	Diagnostic	France	Cross-Sectional	MSK	40 (5-fold cross validation)	DL	CNN [inspired by Garcia & Delakis]	No	No

REFERENCE

1 Zhong Y, She Y, Deng J et al (2022) Deep Learning for Prediction of N2 Metastasis and Survival for Clinical Stage I Non-Small Cell Lung Cancer. Radiology 302:200-211

2 Zhang XQ, Zheng RQ, Jin JY, Wang JF, Zhang T, Zeng J (2022) US Shear-Wave Elastography Dispersion for Characterization of Chronic Liver Disease. Radiology 305:597-605

3 Zhang M, Wong SW, Wright JN et al (2022) MRI Radiogenomics of Pediatric Medulloblastoma: A Multicenter Study. Radiology 304:406-416

4 You S, Masutani EM, Alley MT et al (2022) Deep Learning Automated Background Phase Error Correction for Abdominopelvic 4D Flow MRI. Radiology 302:584-592

5 Wanders AJT, Mees W, Bun PAM et al (2022) Interval Cancer Detection Using a Neural Network and Breast Density in Women with Negative Screening Mammograms. Radiology 303:269-275

6 Verburg E, van Gils CH, van der Velden BHM et al (2022) Deep Learning for Automated Triaging of 4581 Breast MRI Examinations from the DENSE Trial. Radiology 302:29-36

7 Tallam H, Elton DC, Lee S, Wakim P, Pickhardt PJ, Summers RM (2022) Fully Automated Abdominal CT Biomarkers for Type 2 Diabetes Using Deep Learning. Radiology 304:85-95

8 Swinburne NC, Yadav V, Kim J et al (2022) Semisupervised Training of a Brain MRI Tumor Detection Model Using Mined Annotations. Radiology 303:80-89

9 Shoshan Y, Bakalo R, Gilboa-Solomon F et al (2022) Artificial Intelligence for Reducing Workload in Breast Cancer Screening with Digital Breast Tomosynthesis. Radiology 303:69-77

10 Shin J, Seo N, Baek SE et al (2022) MRI Radiomics Model Predicts Pathologic Complete Response of Rectal Cancer Following Chemoradiotherapy. Radiology 303:351-358

11 Sellergren AB, Chen C, Nabulsi Z et al (2022) Simplified Transfer Learning for Chest Radiography Models Using Less Data. Radiology 305:454-465

12 Ramtohul T, Tescher C, Vaflard P et al (2022) Prospective Evaluation of Ultrafast Breast MRI for Predicting Pathologic Response after Neoadjuvant Therapies. Radiology 305:565-574

13 Pezel T, Ambale-Venkatesh B, Quinaglia T et al (2022) Change in Left Atrioventricular Coupling Index to Predict Incident Atrial Fibrillation: The Multi-Ethnic Study of Atherosclerosis (MESA). Radiology 303:317-326

14 Pease M, Arefan D, Barber J et al (2022) Outcome Prediction in Patients with Severe Traumatic Brain Injury Using Deep Learning from Head CT Scans. Radiology 304:385-394

15 Nishii T, Kobayashi T, Tanaka H et al (2022) Deep Learning-based Post Hoc CT Denoising for Myocardial Delayed Enhancement. Radiology 305:82-91

16 Nam JG, Kang HR, Lee SM et al (2022) Deep Learning Prediction of Survival in Patients with Chronic Obstructive Pulmonary Disease Using Chest Radiographs. Radiology 305:199-208

17 Mu D, Bai J, Chen W et al (2022) Calcium Scoring at Coronary CT Angiography Using Deep Learning. Radiology 302:309-316

18 Mohajer B, Dolatshahi M, Moradi K et al (2022) Role of Thigh Muscle Changes in Knee Osteoarthritis Outcomes: Osteoarthritis Initiative Data. Radiology 305:169-178

19 Lee SE, Kim GR, Han K et al (2022) US, Mammography, and Histopathologic Evaluation to Identify Low Nuclear Grade Ductal Carcinoma in Situ. Radiology 303:276-284

20 Lee SA, Lee Y, Ryu HS et al (2022) Diffusion-weighted Breast MRI in Prediction of Upstaging in Women with Biopsy-proven Ductal Carcinoma in Situ. Radiology 305:307-316

21 Kwon BR, Shin SU, Kim SY et al (2022) Microcalcifications and Peritumoral Edema Predict Survival Outcome in Luminal Breast Cancer Treated with Neoadjuvant Chemotherapy. Radiology 304:310-319

22 Krishnan AP, Song Z, Clayton D et al (2022) Joint MRI T1 Unenhancing and Contrast-enhancing Multiple Sclerosis Lesion Segmentation with Deep Learning in OPERA Trials. Radiology 302:662-673

23 Jaramillo D, Duong P, Nguyen JC et al (2022) Diffusion Tensor Imaging of the Knee to Predict Childhood Growth. Radiology 303:655-663

24 Hou R, Grimm LJ, Mazurowski MA et al (2022) Prediction of Upstaging in Ductal Carcinoma in Situ Based on Mammographic Radiomic Features. Radiology 303:54-62

25 Fan M, Cui Y, You C et al (2022) Radiogenomic Signatures of Oncotype DX Recurrence Score Enable Prediction of Survival in Estrogen Receptor-Positive Breast Cancer: A Multicohort Study. Radiology 302:516-524

26 Choe J, Hwang HJ, Seo JB et al (2022) Content-based Image Retrieval by Using Deep Learning for Interstitial Lung Disease Diagnosis with Chest CT. Radiology 302:187-197

27 Chen Y, Gao Z, He Y et al (2022) An Artificial Intelligence Model Based on ACR TI-RADS Characteristics for US Diagnosis of Thyroid Nodules. Radiology 303:613-619

28 Chen JV, Chaudhari G, Hess CP et al (2022) Deep Learning to Predict Neonatal and Infant Brain Age from Myelination on Brain MRI Scans. Radiology 305:678-687

29 Chen H, Yang BW, Qian L et al (2022) Deep Learning Prediction of Ovarian Malignancy at US Compared with O-RADS and Expert Assessment. Radiology 304:106-113

30 Bressem KK, Adams LC, Proft F et al (2023) Deep Learning Detects Changes Indicative of Axial Spondyloarthritis at MRI of Sacroiliac Joints. Radiology 307:e239007

31 Benzakoun J, Deslys MA, Legrand L et al (2022) Synthetic FLAIR as a Substitute for FLAIR Sequence in Acute Ischemic Stroke. Radiology 303:153-159

32 Aquino GJ, Abadia AF, Schoepf UJ et al (2022) Coronary CT Fractional Flow Reserve before Transcatheter Aortic Valve Replacement: Clinical Outcomes. Radiology 302:50-58

33 Alabed S, Alandejani F, Dwivedi K et al (2022) Validation of Artificial Intelligence Cardiac MRI Measurements: Relationship to Heart Catheterization and Mortality Prediction. Radiology 305:68-79

34 Yao F, Bian S, Zhu D et al (2022) Machine learning-based radiomics for multiple primary prostate cancer biological characteristics prediction with (18)F-PSMA-1007 PET: comparison among different volume segmentation thresholds. Radiol Med 127:1170-1178

35 Xue K, Liu L, Liu Y, Guo Y, Zhu Y, Zhang M (2022) Radiomics model based on multi-sequence MR images for predicting preoperative immunoscore in rectal cancer. Radiol Med 127:702-713

36 Wang FH, Zheng HL, Li JT et al (2022) Prediction of recurrence-free survival and adjuvant therapy benefit in patients with gastrointestinal stromal tumors based on radiomics features. Radiol Med 127:1085-1097

37 van der Lubbe M, Vaidyanathan A, de Wit M et al (2022) A non-invasive, automated diagnosis of Meniere's disease using radiomics and machine learning on conventional magnetic resonance imaging: A multicentric, case-controlled feasibility study. Radiol Med 127:72-82

38 Song W, Chen Q, Guo D, Jiang C (2022) Preoperative estimation of the survival of patients with unresectable hepatocellular carcinoma achieving complete response after conventional transcatheter arterial chemoembolization: assessments of clinical and LI-RADS MR features. Radiol Med 127:939-949

39 Palmisano A, Vignale D, Boccia E et al (2022) AI-SCoRE (artificial intelligence-SARS CoV2 risk evaluation): a fast, objective and fully automated platform to predict the outcome in COVID-19 patients. Radiol Med 127:960-972

40 Han D, Yu N, Yu Y, He T, Duan X (2022) Performance of CT radiomics in predicting the overall survival of patients with stage III clear cell renal carcinoma after radical nephrectomy. Radiol Med 127:837-847

41 Gregucci F, Fiorentino A, Mazzola R et al (2022) Radiomic analysis to predict local response in locally advanced pancreatic cancer treated with stereotactic body radiation therapy. Radiol

Med 127:100-107
42 Granata V, Fusco R, De Muzio F et al (2022) Radiomics textural features by MR imaging to assess clinical outcomes following liver resection in colorectal liver metastases. Radiol Med 127:461-470
43 Granata V, Fusco R, De Muzio F et al (2022) Radiomics and machine learning analysis based on magnetic resonance imaging in the assessment of liver mucinous colorectal metastases. Radiol Med 127:763-772
44 Gitto S, Bologna M, Corino VDA et al (2022) Diffusion-weighted MRI radiomics of spine bone tumors: feature stability and machine learning-based classification performance. Radiol Med 127:518-525
45 Gao W, Wang W, Song D et al (2022) A predictive model integrating deep and radiomics features based on gadobenate dimeglumine-enhanced MRI for postoperative early recurrence of hepatocellular carcinoma. Radiol Med 127:259-271
46 Fan Y, Zhao Z, Wang X et al (2022) Radiomics for prediction of response to EGFR-TKI based on metastasis/brain parenchyma (M/BP)-interface. Radiol Med 127:1342-1354
47 Cilla S, Macchia G, Lenkowicz J et al (2022) CT angiography-based radiomics as a tool for carotid plaque characterization: a pilot study. Radiol Med 127:743-753
48 Chiti G, Grazzini G, Flammia F et al (2022) Gastroenteropancreatic neuroendocrine neoplasms (GEP-NENs): a radiomic model to predict tumor grade. Radiol Med 127:928-938
49 Chiloiro G, Cusumano D, de Franco P et al (2022) Does restaging MRI radiomics analysis improve pathological complete response prediction in rectal cancer patients? A prognostic model development. Radiol Med 127:11-20
50 Caruso D, Polici M, Rinzivillo M et al (2022) CT-based radiomics for prediction of therapeutic response to Everolimus in metastatic neuroendocrine tumors. Radiol Med 127:691-701
51 Calandrelli R, Boldrini L, Tran HE et al (2022) CT-based radiomics modeling for skull dysmorphology severity and surgical outcome prediction in children with isolated sagittal synostosis: a hypothesis-generating study. Radiol Med 127:616-626
52 Autorino R, Gui B, Panza G et al (2022) Radiomics-based prediction of two-year clinical outcome in locally advanced cervical cancer patients undergoing neoadjuvant chemoradiotherapy. Radiol Med 127:498-506
53 Abdullah SS, Rajasekaran MP (2022) Automatic detection and classification of knee osteoarthritis using deep learning approach. Radiol Med 127:398-406
54 Ziegelmayer S, Reischl S, Harder F, Makowski M, Braren R, Gawlitza J (2022) Feature Robustness and Diagnostic Capabilities of Convolutional Neural Networks Against Radiomics Features in Computed Tomography Imaging. Invest Radiol 57:171-177
55 Wennmann M, Klein A, Bauer F et al (2022) Combining Deep Learning and Radiomics for Automated, Objective, Comprehensive Bone Marrow Characterization From Whole-Body MRI: A Multicentric Feasibility Study. Invest Radiol 57:752-763
56 Toda N, Hashimoto M, Arita Y et al (2022) Deep Learning Algorithm for Fully Automated Detection of Small (</=4 cm) Renal Cell Carcinoma in Contrast-Enhanced Computed Tomography Using a Multicenter Database. Invest Radiol 57:327-333
57 Thomas MF, Kofler F, Grundl L et al (2022) Improving Automated Glioma Segmentation in Routine Clinical Use Through Artificial Intelligence-Based Replacement of Missing Sequences With Synthetic Magnetic Resonance Imaging Scans. Invest Radiol 57:187-193

58 Storelli L, Azzimonti M, Gueye M et al (2022) A Deep Learning Approach to Predicting Disease Progression in Multiple Sclerosis Using Magnetic Resonance Imaging. Invest Radiol 57:423-432
59 Sexauer R, Yang S, Weikert T et al (2022) Automated Detection, Segmentation, and Classification of Pleural Effusion From Computed Tomography Scans Using Machine Learning. Invest Radiol 57:552-559
60 Rudolph J, Huemmer C, Ghesu FC et al (2022) Artificial Intelligence in Chest Radiography Reporting Accuracy: Added Clinical Value in the Emergency Unit Setting Without 24/7 Radiology Coverage. Invest Radiol 57:90-98
61 Lee SB, Cho YJ, Hong Y et al (2022) Deep Learning-Based Image Conversion Improves the Reproducibility of Computed Tomography Radiomics Features: A Phantom Study. Invest Radiol 57:308-317
62 Huber FA, Chaitanya K, Gross N et al (2022) Whole-body Composition Profiling Using a Deep Learning Algorithm: Influence of Different Acquisition Parameters on Algorithm Performance and Robustness. Invest Radiol 57:33-43
63 Haubold J, Jost G, Theysohn JM et al (2022) Contrast Media Reduction in Computed Tomography With Deep Learning Using a Generative Adversarial Network in an Experimental Animal Study. Invest Radiol 57:696-703
64 Ammari S, Bone A, Balleyguier C et al (2022) Can Deep Learning Replace Gadolinium in Neuro-Oncology?: A Reader Study. Invest Radiol 57:99-107
65 Afat S, Wessling D, Afat C et al (2022) Analysis of a Deep Learning-Based Superresolution Algorithm Tailored to Partial Fourier Gradient Echo Sequences of the Abdomen at 1.5 T: Reduction of Breath-Hold Time and Improvement of Image Quality. Invest Radiol 57:157-162
66 Zou M, Wu D, Zhu H et al (2022) Multiparametric quantitative MRI for the evaluation of dysthyroid optic neuropathy. Eur Radiol 32:1931-1938
67 Zhu C, Hu J, Wang X et al (2022) A novel clinical radiomics nomogram at baseline to predict mucosal healing in Crohn's disease patients treated with infliximab. Eur Radiol 32:6628-6636
68 Zhou Y, Zhou G, Zhang J, Xu C, Zhu F, Xu P (2022) DCE-MRI based radiomics nomogram for preoperatively differentiating combined hepatocellular-cholangiocarcinoma from mass-forming intrahepatic cholangiocarcinoma. Eur Radiol 32:5004-5015
69 Zhou Y, Gu HL, Zhang XL, Tian ZF, Xu XQ, Tang WW (2022) Multiparametric magnetic resonance imaging-derived radiomics for the prediction of disease-free survival in early-stage squamous cervical cancer. Eur Radiol 32:2540-2551
70 Zhou JY, Shi YB, Xia C et al (2022) Beyond collaterals: brain frailty additionally improves prediction of clinical outcome in acute ischemic stroke. Eur Radiol 32:6943-6952
71 Zhong J, Zhang C, Hu Y et al (2022) Automated prediction of the neoadjuvant chemotherapy response in osteosarcoma with deep learning and an MRI-based radiomics nomogram. Eur Radiol 32:6196-6206
72 Zheng YM, Yuan MG, Zhou RQ et al (2022) A computed tomography-based radiomics signature for predicting expression of programmed death ligand 1 in head and neck squamous cell carcinoma. Eur Radiol 32:5362-5370
73 Zheng Y, Zhou D, Liu H, Wen M (2022) CT-based radiomics analysis of different machine learning models for differentiating benign and malignant parotid tumors. Eur Radiol 32:6953-6964
74 Zheng H, Miao Q, Liu Y et al (2022) Multiparametric MRI-based radiomics model to predict pelvic lymph node invasion for patients with prostate cancer. Eur Radiol 32:5688-5699

75 Zhao M, Kluge K, Papp L et al (2022) Multi-lesion radiomics of PET/CT for non-invasive survival stratification and histologic tumor risk profiling in patients with lung adenocarcinoma. Eur Radiol 32:7056-7067

76 Zhang Q, Guo J, Ouyang H, Chen S, Zhao X, Yu X (2022) Added-value of dynamic contrast-enhanced MRI on prediction of tumor recurrence in locally advanced cervical cancer treated with chemoradiotherapy. Eur Radiol 32:2529-2539

77 Zhang MZ, Ou-Yang HQ, Liu JF et al (2022) Predicting postoperative recovery in cervical spondylotic myelopathy: construction and interpretation of T(2)(*)-weighted radiomic-based extra trees models. Eur Radiol 32:3565-3575

78 Zhang K, Xie SS, Li WC, Ye ZX, Shen ZW, Shen W (2022) Prediction of microvascular invasion in HCC by a scoring model combining Gd-EOB-DTPA MRI and biochemical indicators. Eur Radiol 32:4186-4197

79 Zhang J, Wang G, Ren J et al (2022) Multiparametric MRI-based radiomics nomogram for preoperative prediction of lymphovascular invasion and clinical outcomes in patients with breast invasive ductal carcinoma. Eur Radiol 32:4079-4089

80 Zhang H, Zou Y, Tian F et al (2022) Dual-energy CT may predict post-operative recurrence in early-stage glottic laryngeal cancer: a novel nomogram and risk stratification system. Eur Radiol 32:1921-1930

81 Zhang H, Meng Y, Li Q et al (2022) Two nomograms for differentiating mass-forming chronic pancreatitis from pancreatic ductal adenocarcinoma in patients with chronic pancreatitis. Eur Radiol 32:6336-6347

82 Zhang G, Wu Z, Zhang X et al (2022) CT-based radiomics to predict muscle invasion in bladder cancer. Eur Radiol 32:3260-3268

83 Yu Y, Fan Y, Wang X et al (2022) Gd-EOB-DTPA-enhanced MRI radiomics to predict vessels encapsulating tumor clusters (VETC) and patient prognosis in hepatocellular carcinoma. Eur Radiol 32:959-970

84 Yoo HJ, Kim YJ, Hong H, Hong SH, Chae HD, Choi JY (2022) Deep learning-based fully automated body composition analysis of thigh CT: comparison with DXA measurement. Eur Radiol 32:7601-7611

85 Yang X, Liu M, Ren Y et al (2022) Using contrast-enhanced CT and non-contrast-enhanced CT to predict EGFR mutation status in NSCLC patients-a radiomics nomogram analysis. Eur Radiol 32:2693-2703

86 Yang SS, Wu YS, Pang YJ et al (2022) Development and validation of radiologic scores for guiding individualized induction chemotherapy in T3N1M0 nasopharyngeal carcinoma. Eur Radiol 32:3649-3660

87 Yan C, Wang L, Lin J et al (2022) A fully automatic artificial intelligence-based CT image analysis system for accurate detection, diagnosis, and quantitative severity evaluation of pulmonary tuberculosis. Eur Radiol 32:2188-2199

88 Xu Z, Ding Y, Zhao K et al (2022) MRI characteristics of breast edema for assessing axillary lymph node burden in early-stage breast cancer: a retrospective bicentric study. Eur Radiol 32:8213-8225

89 Xu Y, Lu L, Sun SH et al (2022) Effect of CT image acquisition parameters on diagnostic performance of radiomics in predicting malignancy of pulmonary nodules of different sizes. Eur

Radiol 32:1517-1527
90 Xu XQ, Shen GC, Ma G et al (2022) Prognostic value of post-treatment fluid-attenuated inversion recovery vascular hyperintensity in ischemic stroke after endovascular thrombectomy. Eur Radiol 32:8067-8076
91 Xu H, Liu J, Chen Z et al (2022) Intratumoral and peritumoral radiomics based on dynamic contrast-enhanced MRI for preoperative prediction of intraductal component in invasive breast cancer. Eur Radiol 32:4845-4856
92 Xie X, Yang L, Zhao F et al (2022) A deep learning model combining multimodal radiomics, clinical and imaging features for differentiating ocular adnexal lymphoma from idiopathic orbital inflammation. Eur Radiol 32:6922-6932
93 Xie T, Zhao Q, Fu C, Grimm R, Gu Y, Peng W (2022) Improved value of whole-lesion histogram analysis on DCE parametric maps for diagnosing small breast cancer (</= 1 cm). Eur Radiol 32:1634-1643
94 Wu S, Zhang X, Rui W et al (2022) A nomogram strategy for identifying the subclassification of IDH mutation and ATRX expression loss in lower-grade gliomas. Eur Radiol 32:3187-3198
95 Wu S, Ren Y, Lin X, Huang Z, Zheng Z, Zhang X (2022) Development and validation of a composite AI model for the diagnosis of levator ani muscle avulsion. Eur Radiol 32:5898-5906
96 Wu K, Wu P, Yang K et al (2022) A comprehensive texture feature analysis framework of renal cell carcinoma: pathological, prognostic, and genomic evaluation based on CT images. Eur Radiol 32:2255-2265
97 Wu C, Meng G, Lian J et al (2022) A multi-stage ensemble network system to diagnose adolescent idiopathic scoliosis. Eur Radiol 32:5880-5889
98 Wood DA, Kafiabadi S, Al Busaidi A et al (2022) Deep learning to automate the labelling of head MRI datasets for computer vision applications. Eur Radiol 32:725-736
99 Wesp P, Grosu S, Graser A et al (2022) Deep learning in CT colonography: differentiating premalignant from benign colorectal polyps. Eur Radiol 32:4749-4759
100 Wang Y, Zhu GQ, Zhou CW, Li N, Yang C, Zeng MS (2022) Risk stratification of LI-RADS M and LI-RADS 4/5 combined hepatocellular cholangiocarcinoma: prognostic values of MR imaging features and clinicopathological factors. Eur Radiol 32:5166-5178
101 Wang Y, Lang J, Zuo JZ et al (2022) The radiomic-clinical model using the SHAP method for assessing the treatment response of whole-brain radiotherapy: a multicentric study. Eur Radiol 32:8737-8747
102 Wang S, Sun Y, Li R et al (2022) Diagnostic performance of perilesional radiomics analysis of contrast-enhanced mammography for the differentiation of benign and malignant breast lesions. Eur Radiol 32:639-649
103 Wang R, Jiao Z, Yang L et al (2022) Artificial intelligence for prediction of COVID-19 progression using CT imaging and clinical data. Eur Radiol 32:205-212
104 Wang R, He Y, Xing H et al (2022) Inclusion of quantitative high-density plaque in coronary computed tomographic score system to predict the time of guidewire crossing chronic total occlusion. Eur Radiol 32:4565-4573
105 Wang Q, Chen H, Luo G et al (2022) Performance of novel deep learning network with the incorporation of the automatic segmentation network for diagnosis of breast cancer in automated breast ultrasound. Eur Radiol 32:7163-7172
106 Wang L, Chang L, Luo R et al (2022) An artificial intelligence system using maximum intensity projection MR images facilitates classification of non-mass enhancement breast lesions. Eur

Radiol 32:4857-4867
107 Wang J, Jiang J, Zhang D et al (2022) An integrated AI model to improve diagnostic accuracy of ultrasound and output known risk features in suspicious thyroid nodules. Eur Radiol 32:2120-2129
108 Wang J, Guo K, Cui B, Hou Y, Zhao G, Lu J (2022) Individual [(18)F]FDG PET and functional MRI based on simultaneous PET/MRI may predict seizure recurrence after temporal lobe epilepsy surgery. Eur Radiol 32:3880-3888
109 Wang H, Guo Y, Yan B et al (2022) Development and validation of a prediction model based on clinical and CT features for invasiveness of K. pneumoniae liver abscess. Eur Radiol 32:6397-6406
110 Wackenthaler A, Moliere S, Artzner T et al (2022) Pre-operative CT scan helps predict outcome after liver transplantation for acute-on-chronic grade 3 liver failure. Eur Radiol 32:12-21
111 von Schacky CE, Wilhelm NJ, Schafer VS et al (2022) Development and evaluation of machine learning models based on X-ray radiomics for the classification and differentiation of malignant and benign bone tumors. Eur Radiol 32:6247-6257
112 Visser JJ, de Vries M, Kors JA (2022) Automatic detection of actionable findings and communication mentions in radiology reports using natural language processing. Eur Radiol 32:3996-4002
113 Uhlig J, Uhlig A, Bachanek S et al (2022) Primary renal sarcomas: imaging features and discrimination from non-sarcoma renal tumors. Eur Radiol 32:981-989
114 Tzanis E, Damilakis J (2022) A novel methodology to train and deploy a machine learning model for personalized dose assessment in head CT. Eur Radiol 32:6418-6426
115 Tsuchiya M, Masui T, Terauchi K et al (2022) MRI-based radiomics analysis for differentiating phyllodes tumors of the breast from fibroadenomas. Eur Radiol 32:4090-4100
116 Tran A, Lassalle L, Zille P et al (2022) Deep learning to detect anterior cruciate ligament tear on knee MRI: multi-continental external validation. Eur Radiol 32:8394-8403
117 Tomita H, Kobayashi T, Takaya E et al (2022) Deep learning approach of diffusion-weighted imaging as an outcome predictor in laryngeal and hypopharyngeal cancer patients with radiotherapy-related curative treatment: a preliminary study. Eur Radiol 32:5353-5361
118 Ternifi R, Wang Y, Gu J et al (2022) Ultrasound high-definition microvasculature imaging with novel quantitative biomarkers improves breast cancer detection accuracy. Eur Radiol 32:7448-7462
119 Tang WJ, Kong QC, Cheng ZX et al (2022) Performance of radiomics models for tumour-infiltrating lymphocyte (TIL) prediction in breast cancer: the role of the dynamic contrast-enhanced (DCE) MRI phase. Eur Radiol 32:864-875
120 Tang CX, Qiao HY, Zhang XL et al (2022) Functional CAD-RADS using FFR(CT) on therapeutic management and prognosis in patients with coronary artery disease. Eur Radiol 32:5210-5221
121 Sushentsev N, Rundo L, Blyuss O et al (2022) Comparative performance of MRI-derived PRECISE scores and delta-radiomics models for the prediction of prostate cancer progression in patients on active surveillance. Eur Radiol 32:680-689
122 Sun X, Ge J, Li L et al (2022) Use of deep learning-based radiomics to differentiate Parkinson's disease patients from normal controls: a study based on [(18)F]FDG PET imaging. Eur Radiol 32:8008-8018

123 Sun SH, Eche T, Dorczynski C et al (2022) Predicting death or recurrence of portal hypertension symptoms after TIPS procedures. Eur Radiol 32:3346-3357

124 Sun J, Liao X, Yan Y et al (2022) Detection and staging of chronic obstructive pulmonary disease using a computed tomography-based weakly supervised deep learning approach. Eur Radiol 32:5319-5329

125 Song SE, Cho KR, Cho Y et al (2022) Machine learning with multiparametric breast MRI for prediction of Ki-67 and histologic grade in early-stage luminal breast cancer. Eur Radiol 32:853-863

126 Skarping I, Larsson M, Fornvik D (2022) Analysis of mammograms using artificial intelligence to predict response to neoadjuvant chemotherapy in breast cancer patients: proof of concept. Eur Radiol 32:3131-3141

127 Sieren MM, Widmann C, Weiss N et al (2022) Automated segmentation and quantification of the healthy and diseased aorta in CT angiographies using a dedicated deep learning approach. Eur Radiol 32:690-701

128 Si N, Shi K, Li N et al (2022) Identification of patients with acute myocardial infarction based on coronary CT angiography: the value of pericoronary adipose tissue radiomics. Eur Radiol 32:6868-6877

129 Shu Z, Mao D, Song Q, Xu Y, Pang P, Zhang Y (2022) Multiparameter MRI-based radiomics for preoperative prediction of extramural venous invasion in rectal cancer. Eur Radiol 32:1002-1013

130 Sheng R, Huang X, Jin K et al (2022) Contrast-enhanced MRI could predict response of systemic therapy in advanced intrahepatic cholangiocarcinoma. Eur Radiol 32:5156-5165

131 Sheng DL, Shen XG, Shi ZT, Chang C, Li JW (2022) Survival outcome assessment for triple-negative breast cancer: a nomogram analysis based on integrated clinicopathological, sonographic, and mammographic characteristics. Eur Radiol 32:6575-6587

132 Rui W, Qiao N, Wu Y et al (2022) Radiomics analysis allows for precise prediction of silent corticotroph adenoma among non-functioning pituitary adenomas. Eur Radiol 32:1570-1578

133 Ruhling S, Navarro F, Sekuboyina A et al (2022) Automated detection of the contrast phase in MDCT by an artificial neural network improves the accuracy of opportunistic bone mineral density measurements. Eur Radiol 32:1465-1474

134 Rouviere O, Moldovan PC, Vlachomitrou A et al (2022) Combined model-based and deep learning-based automated 3D zonal segmentation of the prostate on T2-weighted MR images: clinical evaluation. Eur Radiol 32:3248-3259

135 Ren J, Yuan Y, Tao X (2022) Histogram analysis of diffusion-weighted imaging and dynamic contrast-enhanced MRI for predicting occult lymph node metastasis in early-stage oral tongue squamous cell carcinoma. Eur Radiol 32:2739-2747

136 Pontillo G, Penna S, Cocozza S et al (2022) Stratification of multiple sclerosis patients using unsupervised machine learning: a single-visit MRI-driven approach. Eur Radiol 32:5382-5391

137 Pfob A, Sidey-Gibbons C, Barr RG et al (2022) The importance of multi-modal imaging and clinical information for humans and AI-based algorithms to classify breast masses (INSPiRED 003): an international, multicenter analysis. Eur Radiol 32:4101-4115

138 Peng WL, Zhang TJ, Shi K et al (2022) Automatic machine learning based on native T1 mapping can identify myocardial fibrosis in patients with hypertrophic cardiomyopathy. Eur Radiol 32:1044-1053

139 Park YW, Kim S, Park CJ et al (2022) Adding radiomics to the 2021 WHO updates may improve prognostic prediction for current IDH-wildtype histological lower-grade gliomas with known EGFR amplification and TERT promoter mutation status. Eur Radiol 32:8089-8098

140 Park YW, Eom J, Kim D et al (2022) A fully automatic multiparametric radiomics model for differentiation of adult pilocytic astrocytomas from high-grade gliomas. Eur Radiol 32:4500-4509

141 Park S, Lee SM, Ahn Y et al (2022) Identification of predictors for brain metastasis in newly diagnosed non-small cell lung cancer: a single-center cohort study. Eur Radiol 32:990-1001

142 Park HY, Suh CH, Shim WH et al (2022) Prognostic value of diffusion-weighted imaging in patients with newly diagnosed sporadic Creutzfeldt-Jakob disease. Eur Radiol 32:1941-1950

143 Park D, Oh D, Lee M et al (2022) Importance of CT image normalization in radiomics analysis: prediction of 3-year recurrence-free survival in non-small cell lung cancer. Eur Radiol 32:8716-8725

144 Ou C, Li C, Qian Y et al (2022) Morphology-aware multi-source fusion-based intracranial aneurysms rupture prediction. Eur Radiol 32:5633-5641

145 Nowak S, Theis M, Wichtmann BD et al (2022) End-to-end automated body composition analyses with integrated quality control for opportunistic assessment of sarcopenia in CT. Eur Radiol 32:3142-3151

146 Noortman WA, Vriens D, de Geus-Oei LF et al (2022) [(18)F]FDG-PET/CT radiomics for the identification of genetic clusters in pheochromocytomas and paragangliomas. Eur Radiol 32:7227-7236

147 Noguchi S, Nishio M, Sakamoto R et al (2022) Deep learning-based algorithm improved radiologists' performance in bone metastases detection on CT. Eur Radiol 32:7976-7987

148 Nagaraj Y, de Jonge G, Andreychenko A et al (2022) Facilitating standardized COVID-19 suspicion prediction based on computed tomography radiomics in a multi-demographic setting. Eur Radiol 32:6384-6396

149 Muller L, Kloeckner R, Mahringer-Kunz A et al (2022) Fully automated AI-based splenic segmentation for predicting survival and estimating the risk of hepatic decompensation in TACE patients with HCC. Eur Radiol 32:6302-6313

150 Meng Y, Ruan J, Yang B et al (2022) Automated quality assessment of chest radiographs based on deep learning and linear regression cascade algorithms. Eur Radiol 32:7680-7690

151 Meng J, Luo Z, Chen Z et al (2022) Intestinal fibrosis classification in patients with Crohn's disease using CT enterography-based deep learning: comparisons with radiomics and radiologists. Eur Radiol 32:8692-8705

152 Matsumoto T, Ehara S, Walston SL, Mitsuyama Y, Miki Y, Ueda D (2022) Artificial intelligence-based detection of atrial fibrillation from chest radiographs. Eur Radiol 32:5890-5897

153 Mao N, Shi Y, Lian C et al (2022) Intratumoral and peritumoral radiomics for preoperative prediction of neoadjuvant chemotherapy effect in breast cancer based on contrast-enhanced spectral mammography. Eur Radiol 32:3207-3219

154 Ma Y, Wang G, Gao F et al (2022) Clinical utility of 3D magnetic resonance elastography in patients with biliary obstruction. Eur Radiol 32:2050-2059

155 Ma Q, Yi Y, Liu T et al (2022) MRI-based radiomics signature for identification of invisible basal cisterns changes in tuberculous meningitis: a preliminary multicenter study. Eur Radiol 32:8659-8669

156 Ma M, Liu R, Wen C et al (2022) Predicting the molecular subtype of breast cancer and identifying interpretable imaging features using machine learning algorithms. Eur Radiol 32:1652-1662

157 Luo X, Piao S, Li H et al (2022) Multi-lesion radiomics model for discrimination of relapsing-remitting multiple sclerosis and neuropsychiatric systemic lupus erythematosus. Eur Radiol 32:5700-5710

158 Luo S, Wei R, Lu S et al (2022) Fuhrman nuclear grade prediction of clear cell renal cell carcinoma: influence of volume of interest delineation strategies on machine learning-based dynamic enhanced CT radiomics analysis. Eur Radiol 32:2340-2350

159 Luo N, Huang X, Ji Y et al (2022) A functional liver imaging score for preoperative prediction of liver failure after hepatocellular carcinoma resection. Eur Radiol 32:5623-5632

160 Ludemann W, Kahn J, Pustelnik D et al (2022) Yttrium-90 radioembolization for unresectable hepatocellular carcinoma: predictive modeling strategies to anticipate tumor response and improve patient selection. Eur Radiol 32:4687-4698

161 Lu SS, Wu RR, Cao YZ et al (2022) ASPECTS-based net water uptake predicts poor reperfusion and poor clinical outcomes in patients with ischemic stroke. Eur Radiol 32:7026-7035

162 Lopes RR, van den Boogert TPW, Lobe NHJ et al (2022) Machine learning-based prediction of insufficient contrast enhancement in coronary computed tomography angiography. Eur Radiol 32:7136-7145

163 Liu Y, Peng C, Chai H et al (2022) Predicting ultrasound-guided thermal ablation benefit in primary hyperparathyroidism. Eur Radiol 32:8497-8506

164 Liu X, Long M, Sun C et al (2022) CT-based radiomics signature analysis for evaluation of response to induction chemotherapy and progression-free survival in locally advanced hypopharyngeal carcinoma. Eur Radiol 32:7755-7766

165 Liu S, Sun W, Yang S et al (2022) Deep learning radiomic nomogram to predict recurrence in soft tissue sarcoma: a multi-institutional study. Eur Radiol 32:793-805

166 Liu R, Pan D, Xu Y et al (2022) A deep learning-machine learning fusion approach for the classification of benign, malignant, and intermediate bone tumors. Eur Radiol 32:1371-1383

167 Liu K, Li K, Wu T et al (2022) Improving the accuracy of prognosis for clinical stage I solid lung adenocarcinoma by radiomics models covering tumor per se and peritumoral changes on CT. Eur Radiol 32:1065-1077

168 Liu J, Guo W, Zeng P et al (2022) Vertebral MRI-based radiomics model to differentiate multiple myeloma from metastases: influence of features number on logistic regression model performance. Eur Radiol 32:572-581

169 Liu B, Zeng Q, Huang J et al (2022) IVIM using convolutional neural networks predicts microvascular invasion in HCC. Eur Radiol 32:7185-7195

170 Lin X, Li Z, Chen S et al (2022) Divergent white matter changes in patients with nasopharyngeal carcinoma post-radiotherapy with different outcomes: a potential biomarker for prediction of radiation necrosis. Eur Radiol 32:7036-7047

171 Lin FY, Chang YC, Huang HY, Li CC, Chen YC, Chen CM (2022) A radiomics approach for lung nodule detection in thoracic CT images based on the dynamic patterns of morphological variation. Eur Radiol 32:3767-3777

172 Lim RP, Kachel S, Villa ADM et al (2022) CardiSort: a convolutional neural network for cross vendor automated sorting of cardiac MR images. Eur Radiol 32:5907-5920

173 Liang H, Guo Y, Chen X et al (2022) Artificial intelligence for stepwise diagnosis and monitoring of COVID-19. Eur Radiol 32:2235-2245

174 Li ZC, Yan J, Zhang S et al (2022) Glioma survival prediction from whole-brain MRI without tumor segmentation using deep attention network: a multicenter study. Eur Radiol 32:5719-5729

175 Li Y, Xie Y, Xu Y, Zhang N, Li G, Ju S (2022) A new scheme of global feature management improved the performance and stability of radiomics model: a study based on CT images of acute

brainstem infarction. Eur Radiol 32:5508-5516
176 Li Y, Wu Y, He J et al (2022) Automatic coronary artery segmentation and diagnosis of stenosis by deep learning based on computed tomographic coronary angiography. Eur Radiol 32:6037-6045
177 Li Y, Wei D, Liu X et al (2022) Molecular subtyping of diffuse gliomas using magnetic resonance imaging: comparison and correlation between radiomics and deep learning. Eur Radiol 32:747-758
178 Li Y, Ren J, Yang JJ et al (2022) MRI-derived radiomics analysis improves the noninvasive pretreatment identification of multimodality therapy candidates with early-stage cervical cancer. Eur Radiol 32:3985-3995
179 Li Y, Chen Y, Zhao R et al (2022) Development and validation of a nomogram based on pretreatment dynamic contrast-enhanced MRI for the prediction of pathologic response after neoadjuvant chemotherapy for triple-negative breast cancer. Eur Radiol 32:1676-1687
180 Li XN, Yin WH, Sun Y et al (2022) Identification of pathology-confirmed vulnerable atherosclerotic lesions by coronary computed tomography angiography using radiomics analysis. Eur Radiol 32:4003-4013
181 Li X, Qi Z, Du H et al (2022) Deep convolutional neural network for preoperative prediction of microvascular invasion and clinical outcomes in patients with HCCs. Eur Radiol 32:771-782
182 Li M, Ren X, Chen X et al (2022) Combining hyperintense FLAIR rim and radiological features in identifying IDH mutant 1p/19q non-codeleted lower-grade glioma. Eur Radiol 32:3869-3879
183 Lee SB, Cho YJ, Yoon SH et al (2022) Automated segmentation of whole-body CT images for body composition analysis in pediatric patients using a deep neural network. Eur Radiol 32:8463-8472
184 Lee JY, Lee KS, Seo BK et al (2022) Radiomic machine learning for predicting prognostic biomarkers and molecular subtypes of breast cancer using tumor heterogeneity and angiogenesis properties on MRI. Eur Radiol 32:650-660
185 Lee JE, Choi SY, Lee MH et al (2022) Differentiating between benign and malignant ampullary strictures: a prediction model using a nomogram based on CT imaging and clinical findings. Eur Radiol 32:7566-7577
186 Laredo C, Solanes A, Renu A et al (2022) Clinical and therapeutic variables may influence the association between infarct core predicted by CT perfusion and clinical outcome in acute stroke. Eur Radiol 32:4510-4520
187 Kruger J, Ostwaldt AC, Spies L et al (2022) Infratentorial lesions in multiple sclerosis patients: intra- and inter-rater variability in comparison to a fully automated segmentation using 3D convolutional neural networks. Eur Radiol 32:2798-2809
188 Koo CW, Williams JM, Liu G et al (2022) Quantitative CT and machine learning classification of fibrotic interstitial lung diseases. Eur Radiol 32:8152-8161
189 Kolossvary M, Bluemke DA, Fishman EK et al (2022) Temporal assessment of lesion morphology on radiological images beyond lesion volumes-a proof-of-principle study. Eur Radiol 32:8748-8760
190 Klontzas ME, Volitakis E, Aydingoz U, Chlapoutakis K, Karantanas AH (2022) Machine learning identifies factors related to early joint space narrowing in dysplastic and non-dysplastic hips.

Eur Radiol 32:542-550
191 Kimura K, Yoshida S, Tsuchiya J et al (2022) Usefulness of texture features of apparent diffusion coefficient maps in predicting chemoradiotherapy response in muscle-invasive bladder cancer. Eur Radiol 32:671-679
192 Kim E, Lee G, Lee SH, Cho H, Lee HY, Park H (2022) Incremental benefits of size-zone matrix-based radiomics features for the prognosis of lung adenocarcinoma: advantage of spatial partitioning on tumor evaluation. Eur Radiol 32:7691-7699
193 Kim C, Lee G, Oh H et al (2022) A deep learning-based automatic analysis of cardiovascular borders on chest radiographs of valvular heart disease: development/external validation. Eur Radiol 32:1558-1569
194 Kikuchi Y, Togao O, Kikuchi K et al (2022) A deep convolutional neural network-based automatic detection of brain metastases with and without blood vessel suppression. Eur Radiol 32:2998-3005
195 Kapsner LA, Ohlmeyer S, Folle L et al (2022) Automated artifact detection in abbreviated dynamic contrast-enhanced (DCE) MRI-derived maximum intensity projections (MIPs) of the breast. Eur Radiol 32:5997-6007
196 Kang JJ, Chen Y, Xu GD et al (2022) Combining quantitative susceptibility mapping to radiomics in diagnosing Parkinson's disease and assessing cognitive impairment. Eur Radiol 32:6992-7003
197 Jung W, Kim J, Ko J, Jeong G, Kim HG (2022) Highly accelerated 3D MPRAGE using deep neural network-based reconstruction for brain imaging in children and young adults. Eur Radiol 32:5468-5479
198 Juan CJ, Lin SC, Li YH et al (2022) Improving interobserver agreement and performance of deep learning models for segmenting acute ischemic stroke by combining DWI with optimized ADC thresholds. Eur Radiol 32:5371-5381
199 Jonske F, Dederichs M, Kim MS et al (2022) Deep Learning-driven classification of external DICOM studies for PACS archiving. Eur Radiol 32:8769-8776
200 Jing X, Wielema M, Cornelissen LJ et al (2022) Using deep learning to safely exclude lesions with only ultrafast breast MRI to shorten acquisition and reading time. Eur Radiol 32:8706-8715
201 Jin X, Li Y, Yan F et al (2022) Automatic coronary plaque detection, classification, and stenosis grading using deep learning and radiomics on computed tomography angiography images: a multi-center multi-vendor study. Eur Radiol 32:5276-5286
202 Jiang YW, Xu XJ, Wang R, Chen CM (2022) Radiomics analysis based on lumbar spine CT to detect osteoporosis. Eur Radiol 32:8019-8026
203 Jiang M, Li CL, Luo XM et al (2022) Radiomics model based on shear-wave elastography in the assessment of axillary lymph node status in early-stage breast cancer. Eur Radiol 32:2313-2325
204 Jiang L, Wang S, Ai Z et al (2022) Development and external validation of a stability machine learning model to identify wake-up stroke onset time from MRI. Eur Radiol 32:3661-3669
205 Jiang C, Huang X, Li A et al (2022) Radiomics signature from [(18)F]FDG PET images for prognosis predication of primary gastrointestinal diffuse large B cell lymphoma. Eur Radiol 32:5730-5741
206 Jiang C, Chen K, Teng Y et al (2022) Deep learning-based tumour segmentation and total metabolic tumour volume prediction in the prognosis of diffuse large B-cell lymphoma patients in

3D FDG-PET images. Eur Radiol 32:4801-4812
207 Jayaprakasam VS, Paroder V, Gibbs P et al (2022) MRI radiomics features of mesorectal fat can predict response to neoadjuvant chemoradiation therapy and tumor recurrence in patients with locally advanced rectal cancer. Eur Radiol 32:971-980
208 Javorszky N, Homonnay B, Gerstenblith G et al (2022) Deep learning-based atherosclerotic coronary plaque segmentation on coronary CT angiography. Eur Radiol 32:7217-7226
209 Jang EB, Kim HS, Park JE et al (2022) Diffuse glioma, not otherwise specified: imaging-based risk stratification achieves histomolecular-level prognostication. Eur Radiol 32:7780-7788
210 Huang LT, Tsai YS, Liou CF et al (2022) Automated Stanford classification of aortic dissection using a 2-step hierarchical neural network at computed tomography angiography. Eur Radiol 32:2277-2285
211 Huang L, Lin W, Xie D et al (2022) Development and validation of a preoperative CT-based radiomic nomogram to predict pathology invasiveness in patients with a solitary pulmonary nodule: a machine learning approach, multicenter, diagnostic study. Eur Radiol 32:1983-1996
212 Huang J, Yang J, Ding J et al (2022) Development and validation of an ultrasound-based prediction model for differentiating between malignant and benign solid pancreatic lesions. Eur Radiol 32:8296-8305
213 Huang J, Xie X, Wu H et al (2022) Development and validation of a combined nomogram model based on deep learning contrast-enhanced ultrasound and clinical factors to predict preoperative aggressiveness in pancreatic neuroendocrine neoplasms. Eur Radiol 32:7965-7975
214 Hou J, Li H, Zeng B et al (2022) MRI-based radiomics nomogram for predicting temporal lobe injury after radiotherapy in nasopharyngeal carcinoma. Eur Radiol 32:1106-1114
215 Hosseinzadeh M, Saha A, Brand P, Slootweg I, de Rooij M, Huisman H (2022) Deep learning-assisted prostate cancer detection on bi-parametric MRI: minimum training data size requirements and effect of prior knowledge. Eur Radiol 32:2224-2234
216 Hinzpeter R, Baumann L, Guggenberger R, Huellner M, Alkadhi H, Baessler B (2022) Radiomics for detecting prostate cancer bone metastases invisible in CT: a proof-of-concept study. Eur Radiol 32:1823-1832
217 Hejduk P, Marcon M, Unkelbach J et al (2022) Fully automatic classification of automated breast ultrasound (ABUS) imaging according to BI-RADS using a deep convolutional neural network. Eur Radiol 32:4868-4878
218 Healy GM, Salinas-Miranda E, Jain R et al (2022) Pre-operative radiomics model for prognostication in resectable pancreatic adenocarcinoma with external validation. Eur Radiol 32:2492-2505
219 He Z, Mao Y, Lu S et al (2022) Machine learning-based radiomics for histological classification of parotid tumors using morphological MRI: a comparative study. Eur Radiol 32:8099-8110
220 Han S, Kim YI, Oh JS et al (2022) Diagnostic and prognostic values of 2-[(18)F]FDG PET/CT in resectable thymic epithelial tumour. Eur Radiol 32:1173-1183
221 Guo X, Wang J, Wang X et al (2022) Diagnosing autism spectrum disorder in children using conventional MRI and apparent diffusion coefficient based deep learning algorithms. Eur Radiol 32:761-770
222 Gu J, Tong T, He C et al (2022) Deep learning radiomics of ultrasonography can predict response to neoadjuvant chemotherapy in breast cancer at an early stage of treatment: a prospective study. Eur Radiol 32:2099-2109

223 Giannakopoulos P, Montandon ML, Herrmann FR et al (2022) Alzheimer resemblance atrophy index, BrainAGE, and normal pressure hydrocephalus score in the prediction of subtle cognitive decline: added value compared to existing MR imaging markers. Eur Radiol 32:7833-7842

224 Ghosh A, Yekeler E, Dalal D, Holroyd A, States L (2022) Whole-tumour apparent diffusion coefficient (ADC) histogram analysis to identify MYCN-amplification in neuroblastomas: preliminary results. Eur Radiol 32:8453-8462

225 Gerson R, Tu W, Abreu-Gomez J et al (2022) Evaluation of the T2-weighted (T2W) adrenal MRI calculator to differentiate adrenal pheochromocytoma from lipid-poor adrenal adenoma. Eur Radiol 32:8247-8255

226 Garrido-Oliver J, Aviles J, Cordova MM et al (2022) Machine learning for the automatic assessment of aortic rotational flow and wall shear stress from 4D flow cardiac magnetic resonance imaging. Eur Radiol 32:7117-7127

227 Frood R, Clark M, Burton C et al (2022) Utility of pre-treatment FDG PET/CT-derived machine learning models for outcome prediction in classical Hodgkin lymphoma. Eur Radiol 32:7237-7247

228 Fan Y, Dong Y, Wang H et al (2022) Development and externally validate MRI-based nomogram to assess EGFR and T790M mutations in patients with metastatic lung adenocarcinoma. Eur Radiol 32:6739-6751

229 Eifer M, Pinian H, Klang E et al (2022) FDG PET/CT radiomics as a tool to differentiate between reactive axillary lymphadenopathy following COVID-19 vaccination and metastatic breast cancer axillary lymphadenopathy: a pilot study. Eur Radiol 32:5921-5929

230 Duan C, Xiong Y, Cheng K et al (2022) Accelerating susceptibility-weighted imaging with deep learning by complex-valued convolutional neural network (ComplexNet): validation in clinical brain imaging. Eur Radiol 32:5679-5687

231 Duan C, Deng H, Xiao S et al (2022) Accelerate gas diffusion-weighted MRI for lung morphometry with deep learning. Eur Radiol 32:702-713

232 Du S, Gao S, Zhao R et al (2022) Contrast-free MRI quantitative parameters for early prediction of pathological response to neoadjuvant chemotherapy in breast cancer. Eur Radiol 32:5759-5772

233 Dot G, Schouman T, Dubois G, Rouch P, Gajny L (2022) Fully automatic segmentation of craniomaxillofacial CT scans for computer-assisted orthognathic surgery planning using the nnU-Net framework. Eur Radiol 32:3639-3648

234 Dong Y, Que L, Jia Q et al (2022) Predicting reintervention after thoracic endovascular aortic repair of Stanford type B aortic dissection using machine learning. Eur Radiol 32:355-367

235 Dong SY, Wang WT, Chen XS et al (2022) Microvascular invasion of small hepatocellular carcinoma can be preoperatively predicted by the 3D quantification of MRI. Eur Radiol 32:4198-4209

236 Dong C, Zheng YM, Li J et al (2022) A CT-based radiomics nomogram for differentiation of squamous cell carcinoma and non-Hodgkin's lymphoma of the palatine tonsil. Eur Radiol 32:243-253

237 Dominique C, Callonnec F, Berghian A et al (2022) Deep learning analysis of contrast-enhanced spectral mammography to determine histoprognostic factors of malignant breast tumours. Eur Radiol 32:4834-4844

238 Demirjian NL, Varghese BA, Cen SY et al (2022) CT-based radiomics stratification of tumor grade and TNM stage of clear cell renal cell carcinoma. Eur Radiol 32:2552-2563

239 Demircioglu A, Quinsten AS, Forsting M, Umutlu L, Nassenstein K (2022) Pediatric age estimation from radiographs of the knee using deep learning. Eur Radiol 32:4813-4822

240 Dana J, Lefebvre TL, Savadjiev P et al (2022) Malignancy risk stratification of cystic renal lesions based on a contrast-enhanced CT-based machine learning model and a clinical decision algorithm. Eur Radiol 32:4116-4127

241 Dai Q, Liu D, Tao Y et al (2022) Nomograms based on preoperative multimodal ultrasound of papillary thyroid carcinoma for predicting central lymph node metastasis. Eur Radiol 32:4596-4608

242 Dai M, Liu Y, Hu Y et al (2022) Combining multiparametric MRI features-based transfer learning and clinical parameters: application of machine learning for the differentiation of uterine sarcomas from atypical leiomyomas. Eur Radiol 32:7988-7997

243 Corrado PA, Wentland AL, Starekova J, Dhyani A, Goss KN, Wieben O (2022) Fully automated intracardiac 4D flow MRI post-processing using deep learning for biventricular segmentation. Eur Radiol 32:5669-5678

244 Chu F, Liu Y, Liu Q et al (2022) Development and validation of MRI-based radiomics signatures models for prediction of disease-free survival and overall survival in patients with esophageal squamous cell carcinoma. Eur Radiol 32:5930-5942

245 Cheng J, Sollee J, Hsieh C et al (2022) COVID-19 mortality prediction in the intensive care unit with deep learning based on longitudinal chest X-rays and clinical data. Eur Radiol 32:4446-4456

246 Cheng B, Deng H, Zhao Y et al (2022) Predicting EGFR mutation status in lung adenocarcinoma presenting as ground-glass opacity: utilizing radiomics model in clinical translation. Eur Radiol 32:5869-5879

247 Chen ZW, Zhao YF, Liu HR et al (2022) Assessment of breast lesions by the Kaiser score for differential diagnosis on MRI: the added value of ADC and machine learning modeling. Eur Radiol 32:6608-6618

248 Chen W, Liu X, Li K et al (2022) A deep-learning model for identifying fresh vertebral compression fractures on digital radiography. Eur Radiol 32:1496-1505

249 Chen M, Yang J, Lu J et al (2022) Ureteral calculi lithotripsy for single ureteral calculi: can DNN-assisted model help preoperatively predict risk factors for sepsis? Eur Radiol 32:8540-8549

250 Chen J, Lu S, Mao Y et al (2022) An MRI-based radiomics-clinical nomogram for the overall survival prediction in patients with hypopharyngeal squamous cell carcinoma: a multi-cohort study. Eur Radiol 32:1548-1557

251 Chen H, Li W, Sheng X et al (2022) Machine learning based on the multimodal connectome can predict the preclinical stage of Alzheimer's disease: a preliminary study. Eur Radiol 32:448-459

252 Chen H, Li S, Zhang Y et al (2022) Deep learning-based automatic segmentation of meningioma from multiparametric MRI for preoperative meningioma differentiation using radiomic features: a multicentre study. Eur Radiol 32:7248-7259

253 Chen C, Qin Y, Chen H et al (2022) Machine learning to differentiate small round cell malignant tumors and non-small round cell malignant tumors of the nasal and paranasal sinuses using apparent diffusion coefficient values. Eur Radiol 32:3819-3829

254 Cha DI, Lee J, Jeong WK et al (2022) Prediction of epithelial-to-mesenchymal transition molecular subtype using CT in gastric cancer. Eur Radiol 32:1-11

255 Cayot B, Milot L, Nempont O et al (2022) Polycystic liver: automatic segmentation using deep learning on CT is faster and as accurate compared to manual segmentation. Eur Radiol 32:4780-4790

256 Cai S, Chen Y, Zhao S et al (2022) Dynamic 3D radiomics analysis using artificial intelligence to assess the stage of COVID-19 on CT images. Eur Radiol 32:4760-4770

257 Brandt V, Schoepf UJ, Aquino GJ et al (2022) Impact of machine-learning-based coronary computed tomography angiography-derived fractional flow reserve on decision-making in patients with severe aortic stenosis undergoing transcatheter aortic valve replacement. Eur Radiol 32:6008-6016

258 Brandt V, Decker J, Schoepf UJ et al (2022) Additive value of epicardial adipose tissue quantification to coronary CT angiography-derived plaque characterization and CT fractional flow reserve for the prediction of lesion-specific ischemia. Eur Radiol 32:4243-4252

259 Bleker J, Kwee TC, Rouw D et al (2022) A deep learning masked segmentation alternative to manual segmentation in biparametric MRI prostate cancer radiomics. Eur Radiol 32:6526-6535

260 Bao D, Zhao Y, Li L et al (2022) A MRI-based radiomics model predicting radiation-induced temporal lobe injury in nasopharyngeal carcinoma. Eur Radiol 32:6910-6921

261 Badic B, Da-Ano R, Poirot K et al (2022) Prediction of recurrence after surgery in colorectal cancer patients using radiomics from diagnostic contrast-enhanced computed tomography: a two-center study. Eur Radiol 32:405-414

262 Aquino GJ, Chamberlin J, Yacoub B et al (2022) Diagnostic accuracy and performance of artificial intelligence in measuring left atrial volumes and function on multiphasic CT in patients with atrial fibrillation. Eur Radiol 32:5256-5264

263 Annovazzi A, Ferraresi V, Rea S et al (2022) Prognostic value of total metabolic tumour volume and therapy-response assessment by [(18)F]FDG PET/CT in patients with metastatic melanoma treated with BRAF/MEK inhibitors. Eur Radiol 32:3398-3407

264 Tao J, Liang C, Yin K et al (2022) 3D convolutional neural network model from contrast-enhanced CT to predict spread through air spaces in non-small cell lung cancer. Diagn Interv Imaging 103:535-544

265 Ren T, Zhang W, Li S et al (2022) Combination of clinical and spectral-CT parameters for predicting lymphovascular and perineural invasion in gastric cancer. Diagn Interv Imaging 103:584-593

266 Paul JF, Rohnean A, Giroussens H, Pressat-Laffouilhere T, Wong T (2022) Evaluation of a deep learning model on coronary CT angiography for automatic stenosis detection. Diagn Interv Imaging 103:316-323

267 Humbert C, Grillet F, Malakhia A et al (2022) Stratification of sigmoid volvulus early recurrence risk using a combination of CT features. Diagn Interv Imaging 103:79-85

268 Fabry V, Mamalet F, Laforet A et al (2022) A deep learning tool without muscle-by-muscle grading to differentiate myositis from facio-scapulo-humeral dystrophy using MRI. Diagn Interv Imaging 103:353-359
